# Stereotypical architecture of the stem cell niche is spatiotemporally established by miR-125-dependent coordination of Notch and steroid signaling

**DOI:** 10.1242/dev.159178

**Published:** 2018-02-01

**Authors:** Andriy S. Yatsenko, Halyna R. Shcherbata

**Affiliations:** Max Planck Research Group of Gene Expression and Signaling, Max Planck Institute for Biophysical Chemistry, Am Fassberg 11, 37077 Göttingen, Germany

**Keywords:** *Drosophila*, Oogenesis, Germline stem cell, Stem cell niche, Cell fate, Steroid, *miR-125*, Tom, Notch, Delta, Notch cell reprogramming

## Abstract

Stem cell niches act as signaling platforms that regulate stem cell self-renewal and sustain stem cells throughout life; however, the specific developmental events controlling their assembly are not well understood. Here, we show that during *Drosophila* ovarian germline stem cell niche formation, the status of Notch signaling in the cell can be reprogrammed. This is controlled via steroid-induced *miR-125*, which targets a negative regulator of Notch signaling, Tom. Thus, *miR-125* acts as a spatiotemporal coordinator between paracrine Notch and endocrine steroid signaling. Moreover, a dual security mechanism for Notch signaling activation exists to ensure the robustness of niche assembly. Particularly, stem cell niche cells can be specified either via lateral inhibition, in which a niche cell precursor acquires Notch signal-sending status randomly, or via peripheral induction, whereby Delta is produced by a specific cell. When one mechanism is perturbed due to mutations, developmental defects or environmental stress, the remaining mechanism ensures that the niche is formed, perhaps abnormally, but still functional. This guarantees that the germline stem cells will have their residence, thereby securing progressive oogenesis and, thus, organism reproduction.

## Abstract (precis)

**Highlighted Article:** In *Drosophila*, the robustness of stem cell niche assembly is safeguarded via a dual mechanism of Notch activation. Cellular Notch status can be reprogrammed by *miR-125*, which spatiotemporally coordinates paracrine and endocrine signaling.

## INTRODUCTION

Stem cells are necessary for the formation and maintenance of any organ. They can sustain their stemness only when located within specialized stem cell niches, which provide the lifelong support, a molecular address and tissue-specific milieu for adult stem cells (Mathieu et al., 2013; Rahman et al., 2017; Rodríguez-Colman et al., 2017). However, our current knowledge about the processes governing stem cell niche assembly in any organism is very limited. Therefore, an essential, but yet not fully understood issue in stem cell biology is how adult stem niches are formed in a developing organ.

**Fig. 1.**
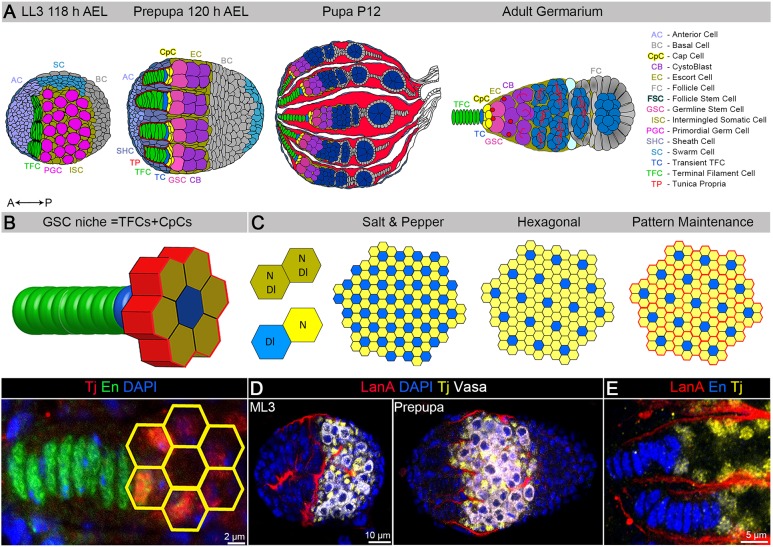
**Patterning of Notch signaling at different stages of ovarian morphogenesis.** (A) The developing *Drosophila* ovary at the late third instar larva (LL3), prepupa, pupa, and adult stages. Different cell types are illustrated by different colors (see the legend on the right). (B) Cartoon of the GSC niche unit, which consists of eight or nine terminal filament cells (TFCs, green; transient TFC, blue) and six cap cells (CpCs, yellow). A, anterior; P, posterior. (C) Schematics of Notch signaling activation in salt-and-pepper and hexagonal patterns, which can be achieved via lateral inhibition or peripheral induction. Undecided cells that co-expresses N and Dl (olive), Notch signal-sending cells (Dl, blue) and Notch signal-receiving cells (N, yellow) are indicated. The hexagonal tessellation requires separation of hexagons to maintain the Notch activity pattern (pattern maintenance). (D,E) The ECM protein LanA (red, LanA::GFP) is present in the tunica propria, which is expressed by SHCs that are separating individual TFs at the prepupal stage. CpCs and ECs are marked by Tj (yellow, D,E), TFCs are marked by En (blue, E), and germline is marked by Vasa (white, D).

A step towards elucidating the molecular mechanisms of stem cell niche formation is analysis of the process in model genetic organisms such as *Drosophila*, which contains one of the best-studied stem cell niches: the ovarian germline stem cell (GSC) niche (Bolívar et al., 2006; Bonfini et al., 2015; Eliazer and Buszczak, 2011; Fuller and Spradling, 2007; Leatherman and Dinardo, 2010; Song et al., 2007; Spradling et al., 2008; Ward et al., 2006). In *Drosophila*, gonad development begins during embryogenesis when primordial germline cells (PGCs) migrate into the abdominal region, where they coalesce with somatic gonad precursor cells to form paired gonads (Asaoka and Lin, 2004; Godt and Laski, 1995; Li et al., 2003; Williamson and Lehmann, 1996). During early postembryonic development, germline and somatic cells remain largely undifferentiated and just undergo mitotic divisions, forming a gonadal primordium (Fig. 1A). At the second instar larval stage [L2, 72 h after egg laying (AEL)], three distinct types of somatic cells can be identified in the developing ovary: apical (ACs), basal (BCs) and intermingled somatic cells (ISCs). During late larval development, at the late third instar larval stage (LL3, 118 h AEL), the formation of the GSC niche begins with terminal filament cell (TFC) differentiation and formation of TF stacks, one per ovariole (Fig. 1A). TF arrangement occurs from medial to lateral across the ovary as a result of progressive differentiation of a group of ACs that are in close proximity to the germline. The number of TFCs in a developing stack gradually increases and then, via cell intercalation, separate TF stalks are formed (Chen et al., 2001; Godt and Laski, 1995). Notably, the number of ovarioles strictly depends on TF number (Hodin and Riddiford, 1998), which in turn is determined by the number of TFC precursors (Sarikaya and Extavour, 2015). At the larval-to-pupal transition (LL3-prepupa, 120 h AEL), some of the ISCs juxtaposed to a TF become the stem cell niche cells (also known as cap cells, CpCs), whereas others develop into escort cells (ECs). ECs form a separate germline differentiation niche while also likely contributing to the GSC niche (Fig. 1A, prepupa, 120 h AEL). Next, PGCs that are adjacent to CpCs become GSCs, while the rest of the germline cells commence differentiation and divide four times with incomplete cytokinesis to produce an oocyte and 15 associated nurse cells (Fig. 1A). At this stage, the stem cell niche unit (Fig. 1B) is made, and the separation of individual ovarioles begins. Importantly, this niche unit is responsible for the maintenance of adult GSCs during the entire lifetime of the animal. It has a highly stereotypical architecture and usually consists of eight or nine TFCs and six CpCs with very little deviation (Fig. 1B). To achieve such a degree of precision in its organization, the process of GSC niche unit formation must be under tight spatiotemporal control.

Previously, multiple signaling pathways governing cell fate during the process of GSC niche assembly have been described (Bonfini et al., 2015; Gancz and Gilboa, 2013; König et al., 2011; Lengil et al., 2015; Lopez-Onieva et al., 2008; Okegbe and DiNardo, 2011; Panchal et al., 2017; Sarikaya and Extavour, 2015; Shimizu et al., 2017; Song et al., 2004), but much remains unclear. In particular, it has been shown that activation of the Notch-Delta (N-Dl) signaling pathway in CpC precursors is essential for their acquisition of GSC niche cell fate (Song et al., 2007; Ward et al., 2006). It has also been shown that the presence of Delta in the posterior TFCs is important for proper niche establishment and that the depletion of Delta in random germline clones does not have a significant effect on niche size (Hsu and Drummond-Barbosa, 2011). However, the complete absence of germline cells results in smaller niches, suggesting that germline signaling influences niche formation (Panchal et al., 2017).

Predominantly, Notch signaling activation occurs as a result of *trans-*interactional communication between two cells, Notch signal-sending and Notch signal-receiving, in which the Notch ligand (Delta or Serrate) has to be delivered to the membrane of the Notch signal-sending cell (Bray, 2006; Lai, 2004). Upon ligand binding to the Notch receptor in the adjacent cell, endocytosis of the Notch-Delta complex by the ligand-expressing cell ensues. This allows Notch receptor cleavage and translocation of the Notch intracellular domain to the nucleus of the Notch signal-receiving cell, thus activating the Notch signaling cascade. In addition, it is known that for Delta activation, several post-translational processing steps are required, e.g. its ubiquitylation by Neuralized (Neur) or inclusion in clathrin-coated vesicles (Bray, 2006; Lai et al., 2001; Le Borgne et al., 2005; Weinmaster and Fischer, 2011; Yeh et al., 2001). Therefore, Notch signaling can be fine-tuned by Bearded family members that act as negative regulators of Neur (Bardin and Schweisguth, 2006; De Renzis et al., 2006; Fontana and Posakony, 2009). In addition to ligand-dependent Notch activation, Notch can be activated independently of its ligands, e.g. via Deltex-induced Notch endocytosis, and this mode of Notch signaling activation also contributes to the regulation of GSC niche size (Shimizu et al., 2017). Here, we have therefore studied in greater detail how the spatiotemporal pattern of Notch signaling activation is coordinated in the developing ovary to secure the precision of stem cell niche assembly.

Notch signaling functions via various *modi operandi* (Lai, 2004). Among a group of equipotent cells, signaling between Notch and Delta can direct binary cell-fate choices: inhibitory Notch signaling that is also called ‘lateral inhibition’ (Barad et al., 2010; Chanet et al., 2009; Fiuza and Arias, 2007; Hunter et al., 2016). Among non-equivalent cell populations, cell fates can be differentially patterned by the strength of Notch activation: inductive Notch signaling or ‘peripheral induction’. In both cases, activation of Notch generates mutually exclusive signaling states between neighboring cells. Therefore, we wanted to identify the physiological sources of Delta that chronologically induce Notch signaling in the niche precursors and via what modes Notch signaling is activated in the process of acquiring niche cell fate by CpCs.

Another key signaling pathway that has an effect on GSC niche formation is steroid hormone 20-hydroxyecdysone (ecdysone) signaling. It has a dual role in the germarium: (1) during development, to regulate the timing of stem cell niche formation, which influences niche size and, subsequently, the number of stem cells these niches can facilitate (Gancz et al., 2011; Hodin and Riddiford, 1998; König et al., 2011); and (2) during adulthood, to maintain the EC fate in the germline differentiation niche, which has a cell non-autonomous effect on the differentiation efficiency of GSC daughters (Fagegaltier et al., 2014; König and Shcherbata, 2015). Thus, previous findings demonstrate that Notch and steroid signaling pathways are involved in the process of ovarian morphogenesis and suggest that these pathways must be coordinated to maintain spatiotemporal precision of niche cell fate specification. Therefore, we wanted to understand whether and how these two essential pathways, paracrine Notch and endocrine ecdysone signaling, interact in the process of stem cell niche morphogenesis.

miRNAs are great candidates to act as intermediaries between crucial signaling pathways, as we have found that they act via complex feedforward and feedback regulatory networks in different tissues, including ovaries (Cicek et al., 2016; Fagegaltier et al., 2014; König and Shcherbata, 2015; Yatsenko et al., 2014; Yatsenko and Shcherbata, 2014). In addition, the miRNA pathway has been shown to play a significant role in the control of GSC self-renewal, and there are developmental stage-specific requirements for miRNAs in this process (Hatfield et al., 2005; Jin and Xie, 2007; Park et al., 2007; Shcherbata et al., 2007; Yang et al., 2016; Yu et al., 2009). However, the role of specific miRNAs in GSC niche formation has not been demonstrated.

Here, we have identified that a single miRNA, *miR-125*, acts as an intermediary between spatial Notch and temporal steroid signaling in the process of stem cell niche establishment. We investigated the role of Notch signaling during ovarian development and found that, in order to specify CpCs, Delta is sent from the TF. Importantly, this requires that the posterior TFC changes its Notch signaling status. As Notch signaling is involved in virtually all biological processes, it is important to recognize that the cellular status regarding Notch activity can be changed. Here, we find that *miR-125*, which is temporally induced by the steroid pulse, targets an antagonist of Notch signaling, Tom. *miR-125* promotes the cell fate reprogramming of the TFC adjacent to the CpC precursors into a Delta-sending cell. This Delta-sending TFC, via peripheral induction, activates Notch signaling in the adjacent GSC niche precursors. It generates a perfect hexagonal pattern of Notch signal-receiving cells, which triggers their terminal differentiation and acquisition of the CpC cell fate for life. Alterations in the expression timing and levels of any of the components of the steroids-*miR-125*-Tom-Delta-Notch signaling cascade cause enlarged or ectopic stem cell niches that can facilitate supernumerary GSCs. The mechanism proposed here of miRNA-based spatiotemporal coordination of essential signaling pathways helps to explain how the precision of ovarian stem cell niche assembly is achieved. A deeper understanding of the processes, factors and principles that govern stem cell niche assembly during development is key for the fields of stem cell biology and regenerative medicine.

## RESULTS

### CpCs originate as a result of hexagonal tiling initiated by Notch activation via peripheral induction

It has been shown previously that activation of Notch signaling in CpC precursors is essential for stem cell niche cell fate (Song et al., 2007; Ward et al., 2006). Importantly, ectopic activation of Notch signaling via Delta overexpression regardless of the source (soma or germline) considerably increases the niche size (Fig. S1A,B) and the number of stem cells it can accommodate (Song et al., 2007; Ward et al., 2006), demonstrating that CpC precursors have the capacity to accept a Notch signal-receiving cell fate irrespective of the tissue from which Delta originates. Notch mutants (*N^1ts^*) fail to form CpCs [note the absence of yellow cells in *N^1ts^* mutant (Fig. S1A,C), suggesting that, in order to become CpCs, ISCs must acquire active Notch signaling status].

Theoretically, in CpC precursors, Notch can be activated even without an external source of Delta, as they co-express Notch and Delta (Fig. S1D,E, olive arrows). According to the rules of lateral inhibition, at least one cell has to shift the balance to express more Notch or Delta, which will immediately initiate the opposite (Delta or Notch) cell fate in the adjacent neighbor. Even in the Petri dish, cells co-expressing Notch and Delta will separate over time into Notch signal-receiving and Notch signal-sending cells (Barad et al., 2010; Matsuda et al., 2015; Petrovic et al., 2014; Sprinzak et al., 2010), assembling into the salt-and-pepper pattern of Notch signaling activity (Fig. 1C). However, as there are always six cap cells around one TFC, this organization does not match the random salt-and-pepper model. Six is the optimal number of cells that can touch one activated cell to generate a regular hexagonal pattern (Fig. 1C), one of the most stable patterns in nature (Doelman and van Harten, 1995). As this patterning is happening at the plane that is located next to the TF that has already been assembled, it allows tissue patterning similar to the tessellation of regular congruent hexagons. Hexagonal tiling is the densest way to arrange round cells in two-dimensional space (Graham et al., 1998). Assuming that this pattern is correct, it requires that each TF becomes the source of Delta, and that the Notch-active CpCs must be separated to maintain the Notch-Notch border (Fig. 1C). It has been documented that Delta from the posterior TFCs affects the formation of CpCs (Hsu and Drummond-Barbosa, 2011), additionally supporting this model. Based on these observations, we propose that CpCs originate as a result of hexagonal tiling initiated by Notch activation via peripheral induction, and at least one of the TFCs must be a Delta-sending cell in order to make the hexagonal pattern possible.

However, there are two discrepancies that do not agree with the established model of Notch signaling to straightforwardly support this model. First, among a group of equipotent cells, such as CpC precursors, Notch signaling usually should work via lateral inhibition, meaning that if one cell is Notch signal sending, then the juxtaposed cell acquires a Notch-receiving fate (Fig. 1C). How is Notch cell fate maintained between different hexagons? It was easy to answer this question because we observed that concurrently with stem cell niche establishment, the tunica propria, the ECM deposited by anterior somatic cells that are moving posteriorly, separates each TF with adjacent CpCs, which precedes individual ovariole formation (Fig. 1D,E).

**Fig. 2.**
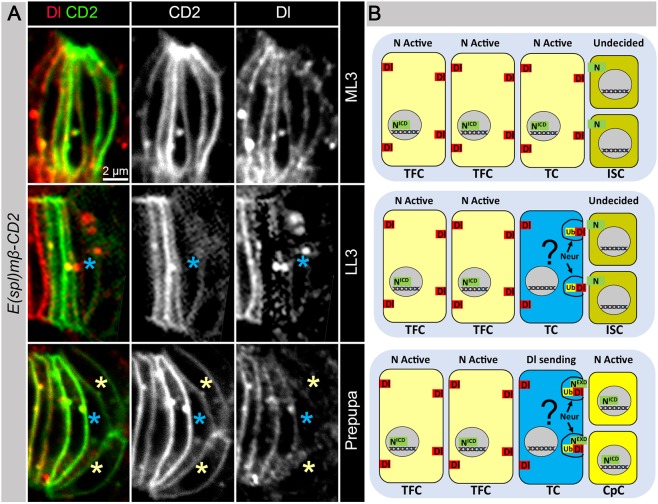
**Reprogramming of the Notch cellular status by posterior TFCs is for proper CpC specification.** (A) Expression of the Notch activity reporter [*E(spl)mβ-CD2*, green] and Dl protein (red) during the GSC niche formation. The Notch activity reporter and Dl protein are expressed in TFCs at ML3 stage. At LL3 stage, levels of the Notch reporter and the Dl protein are reduced in the most posterior TFC (blue asterisks). Dl appears in vesicles, indicating a Notch signal-sending state. At the prepupal stage, the Notch reporter is active in CpCs (yellow asterisks) and levels of the Notch reporter and Dl protein are reduced in the most posterior TFC (TC, blue asterisks). (B) The potential model of TFC reprograming from the Notch active to Dl-sending status.

Second, this model assumes that each TF should serve as a source of Delta. However, when we analyzed Notch activity, using a Notch signaling reporter in which the sequence encoding rat CD2 protein is inserted downstream of the Enhancer of Split [*E(spl)mß*] promoter that is activated by Notch signaling (de Celis et al., 1998), we found that TFCs just prior the CpC specification stage (ML3) have Notch signaling activated (Fig. 2A,B, ML3). Therefore, they cannot send Delta signal and induce Notch signaling in the adjacent CpC precursors, unless one of the TFCs would change its fate and become a Notch signal-sending cell. How can any of them become a Notch signal-sending cell?

To address this question, we carefully examined individual TFs and looked at the Delta protein and Notch activity reporter expression in TFCs in L3 ovaries at the transition when the TF formation is finished (ML3) and CpCs start to differentiate (LL3). In contrast to ML3, it could be clearly seen that some of the posterior TFCs (Fig. 2A,B, LL3, blue asterisk) lose Notch activity and have Delta-positive cytoplasmic vesicles. As a result of Delta activation, the adjacent ISCs acquire Notch active status and terminally differentiate into CpCs (Fig. 2A,B, prepupa, yellow asterisks). Therefore, we next wanted to dissect what activates Delta in the TFCs that allows them to switch their status from Notch signal-receiving to Notch signal-sending and induce hexagonal Notch signaling pattern in CpC precursors (Fig. 2B). As ecdysone and Notch signaling pathways are both required for proper niche establishment (Gancz et al., 2011; Hsu and Drummond-Barbosa, 2011; König et al., 2011; Song et al., 2007; Ward et al., 2006), we hypothesized that these two pathways interact to coordinate the precision of stem cell niche formation and that ecdysone signaling plays a role in TFC reprograming from Notch signal receiving to Notch signal sending.

### Steroid-induced miR-125 targets a Notch signaling factor Tom

**Fig. 3.**
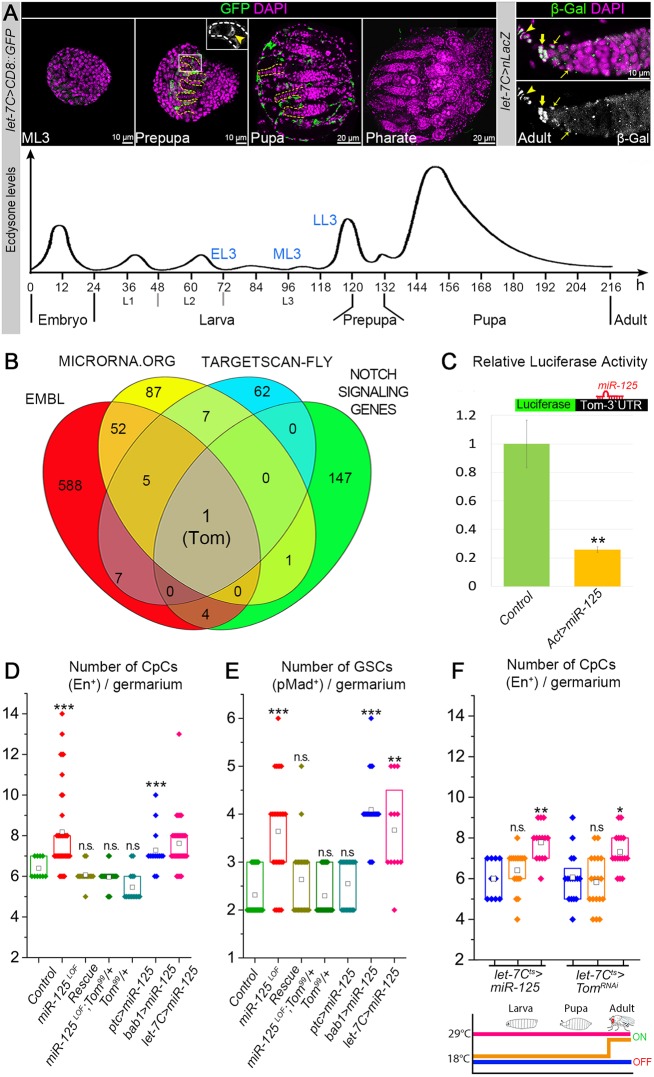
***miR-125* regulates Notch signaling via targeting Tom.** (A) *let-7C* expression (green) at different stages of ovary development. Expression of *let-7C* is detected in solitary TFCs (yellow arrowhead) and SHCs (prepupa-pupa); *let-7C* expression is absent in the pharate ovaries and, later, it can be detected again in the adult germarium in the TFCs and CpCs (high levels), and ECs (low levels). TFs are outlined by dashed lines; thick yellow arrows point to the adult niche cells (CpCs), thin yellow arrows point to ECs. Induction of *let-7C* coincides with the ecdysone pulse at LL3-prepupa stage. (B) Venn diagrams of putative *let-7*, *miR-100* and *miR-125* miRNAs targets from different miRNA target prediction databases (EMBL, microrna.org and TARGETSCAN-Fly) and annotated gene ontology terms for the Notch signaling pathway components (FlyBase). Overlap identifies a single target: Tom. (C) Expression of *miR-125* in S2 cells leads to the downregulation of the *Tom-3′UTR* luciferase reporter. Bar graph represents relative downregulation. Data are mean±s.d. (see also Table S1). (D-F) Box plots represent the number of CpCs, marked by En (D,F), and number of GSCs, marked by pMad, in the adult germaria (E). Loss of *miR-125* (*miR-125^LOF^*) leads to the increased numbers of CpCs and GSCs, whereas reduction of *Tom* levels by one copy in *miR-125* mutants (rescue) restores the normal numbers of CpCs and GSCs. Upregulation of *miR-125* with *bab1-Gal4* and *let-7C-Gal4*, but not with *ptc-Gal4*, drivers increases the CpC and GSC numbers. For *Gal4* driver expression patterns, refer to Fig. S1B-D. (F) *miR-125* upregulation or *Tom* downregulation with *let-7C-Gal4* increases the CpC numbers only when expressed during pre-adult stages but not during adulthood. Colors in the schematics of the gene switch system used for the activation of transgenes corresponds to box plot colors. The box plots (D-F) represent the interquartile range (IQR) or 25th and 75th percentiles of the data set. The mean value (square) is displayed inside the box. One-way ANOVA with post-hoc Tukey HSD was used to test for statistical significance in D-F. All samples were compared with the controls under the same conditions: ***P≤0.001; **P≤0.01; n.s., *P*≥0.05.

Next, we tested whether miRNAs act as conductors, orchestrating paracrine Notch and endocrine steroid signaling in order to ensure the formation of a proper stem cell niche. Ecdysone signaling induces expression of a polycistronic miRNA complex, the *let-7 Complex* (*let-7C*, Fig. S2A), which contains three evolutionarily conserved miRNAs: *miR-100*, *let-7* and *miR-125* (Chawla and Sokol, 2012; Kucherenko et al., 2012; Sempere et al., 2002). Moreover, we have demonstrated that during adult and pre-adult stages, in the processes of neuronal and epithelial cell fate specification, ecdysone signaling robustness is conferred by miRNAs (König et al., 2011; Kucherenko et al., 2012). In addition, ecdysone signaling acts specifically in the soma to regulate the timing of niche cell fate selection by the somatic precursor cells (Gancz et al., 2011; Hodin and Riddiford, 1998). As *let-7C* expression has been shown to be activated by ecdysone signaling (Chawla and Sokol, 2012; Garbuzov and Tatar, 2010; Kucherenko et al., 2012; Sempere et al., 2002), we tested whether *let-7C* is also expressed in the developing ovary and whether its expression parallels the ecdysone pulses (Kozlova and Thummel, 2003) (Fig. 3A). To address this, we analyzed the temporal expression pattern of the GFP protein under the control of the *let-7C* driver (*let-7C-Gal4/UAS-CD8::GFP*). Although at early larval stages we could not detect any GFP signal, it was present at the prepupal stages (Fig. 3A, Fig. S3C). Importantly, the onset of *let-7C* expression in the developing ovaries (LL3-prepupa) coincides with the beginning of CpC specification, as measured by the Notch signaling activation in ISCs (Fig. 2A), suggesting a potential role for the ecdysone-induced *let-7C* miRNAs in niche formation. This pattern is extremely transient; specifically, *let-7C* expression is detected in the solitary TFCs located mostly at the base of the TF and also in single SHCs as they migrate posteriorly between TF stacks, demarcating individual GSC niche units (Fig. 3A, Prepupa, Fig. S3C, arrowheads). At the pharate stage, *let-7C* expression is not detected but then, in the adult germarium, it reappears in the GSC niche (TFCs and CpCs, high levels) and in the differentiation niche (ECs, lower levels) in the germarium (Fig. 3A, pharate and adult). This unique, temporally and spatially defined expression pattern leads us to propose that the niche formation is regulated via one of the three highly evolutionarily conserved, steroid-induced miRNAs of the *let-7* complex – *let-7*, *miR-100* or *miR-125* – and that this miRNA plays a role in the process through a target involved in Notch signaling.

Next, we wanted to determine whether any of the predicted targets of *let-7C* are involved in the regulation of Notch pathway genes. To enhance the probability of finding a bona fide *let-7C* target, we used multiple *in silico* miRNA target prediction databases: TargetScan 6.2 (Kheradpour et al., 2007), miRNA Target Gene Predictions at EMBL (Stark et al., 2003) and miRANDA (microRNA.org) (Enright et al., 2003). Notch-signaling genes were identified using FlyBase annotation database for the GO-term: 0007219 (http://flybase.org/cgi-bin/cvreport.html?rel=is_a&id=GO:0007219). It resulted in a list of genes that were previously annotated with the term Notch signaling pathway, among which only *D. melanogaster* genes were selected. Next, we put together these known Notch signaling regulation genes and compared them with all putative targets of the *let-7* complex miRNAs (Fig. 3B). Among these, only one gene, *Tom*, fulfilled all the requirements: to be predicted as a target by all three target prediction databases and to be previously described as a component of the Notch signaling pathway (Zaffran and Frasch, 2000). *Tom* is a putative target of *miR-125*, but not *miR-100* or *let-7*, suggesting that the ecdysone-induced *miR-125* is a likely candidate to play a role in Notch signaling regulation.

Tom (also known as Twin of m4 or Barbu) is a member of the Bearded family proteins that antagonize Notch signaling via interaction with the E3 ubiquitin ligase Neur, which is required for Delta internalization and, thus, activation (Bardin and Schweisguth, 2006; De Renzis et al., 2006; Fontana and Posakony, 2009; Lai et al., 2000, 2001; Yeh et al., 2001; Zaffran and Frasch, 2000). To validate that Tom is a *miR-125* target*,* we performed *in vitro* luciferase assays in *Drosophila* S2 cells using the reporter that contains the 3′UTR fragment of *Tom* mRNA with the predicted *miR-125*-binding site. Upon overexpression of *miR-125*, the luciferase reporter activity was reduced 2.6-fold (Fig. 3C, Table S1), indicating that *Tom 3*′*UTR* can be targeted by *miR-125*.

### miR-125 affects the process of niche formation

Since the role of *miR-125* and its target Tom in the GSC niche have not been previously studied, we decided to investigate them in more detail. Loss of *miR-125* (*miR-125^LOF^*, Fig. S2C, Table S2) results in significantly higher numbers of the CpCs that stained positively for the GSC niche cell marker Engrailed (En, Fig. 3D, Table S3). To test whether the enlarged niches in *miR-125* mutants are functional and can facilitate higher numbers of GSCs, we quantified the numbers of GSCs per GSC niche unit in the adult germarium using phosphorylated Mad (pMad) as a marker for GSC identity. We found that, in *miR-125* mutants, the number of GSCs is significantly increased, demonstrating that the enlarged GSC niches are fully functional (Fig. 3E, Table S3). To further test whether *miR-125* controls the process of GSC niche formation via its repression of *Tom*, we performed rescue experiments in which we reduced Tom levels by introducing one copy of a Tom mutation (*Tom^99^*) in the *miR-125* mutant background. Analysis of CpC numbers revealed that reducing Tom levels by half is sufficient to fully rescue the *miR-125* mutant niche phenotype (Fig. 3D,E, Table S3), demonstrating that *miR-125*-Tom regulation is required for precise specification of the CpC number in the GSC niche.

Expression analysis of *let-7C* shows that *miR-125* is expressed in TF precursors, whereas its deficiency results in the abnormal numbers of CpCs, suggesting that *miR-125* has a cell non-autonomous effect on CpC specification. To test this hypothesis, we used different *Gal4* drivers to deregulate *miR-125* levels in the different cell types of the developing ovary. In particular, we overexpressed *miR-125* using *ptc-Gal4,* which drives expression in ISCs (Fig. S3A); *bab1-Gal4*, which drives expression in TFCs and CpCs (Fig. S3B); and *let-7C-Gal4*, which is expressed in one solitary cell per TF (Fig. S3C). Analysis of the stem cell niche architecture revealed that, in comparison with controls, overexpression of *miR-125* with *bab1-Gal4* but not with *ptc-Gal4* led to the significant increase of both CpC and GSC numbers (Fig. 3D,E, Table S3). Moreover, *miR-125* overexpression using its own promoter resulted in similar phenotypes, as significantly higher numbers of active CpCs that were able to maintain supernumerary GSCs were observed in mutant germaria (Fig. 3D,E, Table S3). These data show that *miR-125* acts specifically in the TFCs and that it is sufficient to change the expression levels of *miR-125* in the solitary *let-7C*-expressing TFCs to alter the size of the stem cell niche. This implies that *miR-125* has a cell non-autonomous effect on CpC specification.

In addition, we found that in the adult germarium, the *let-*7C promoter is active in all somatic cell types, in TFCs and CpCs of the germline stem cell niche, and in ECs of the germline differentiation niche (Adult, Fig. 3A). Therefore, we needed to exclude that *miR-125* phenotypes are due to the role of *miR-125* in the adult GSC niche. It has been shown that the germline niche cells (TFCs, CpCs and EC) are terminally differentiated cells that do not divide in adulthood (Eliazer et al., 2014; Kirilly et al., 2011; König and Shcherbata, 2015; Morris and Spradling, 2011). Still, there is a possibility that the increased number of CpCs in *miR-125* mutants could appear as a result of atypical CpC proliferation or postdevelopmental EC *trans-*differentiation. To test whether *miR-125* acts by promoting the division of the somatic cells in adult GSC and differentiation niches, we analyzed the presence of the mitosis marker PH3 in the adult germarium. However, in neither control nor mutant flies with *miR-125* misexpression could we observe TFCs, CpCs or ECs that were positive for PH3 (data not shown). Therefore, we concluded that the enlarged niches are not due to atypical divisions of the somatic niche cells in the adult germarium. Even though CpCs are specified only during development, we did not exclude the possibility that the extra CpCs might originate as the result of *trans-*differentiation from other somatic cells (e.g. ECs) in adulthood.

To determine whether *miR-125* is required only during development to regulate GSC niche size or could also affect CpC numbers during adulthood, we used the *Gal80^ts^* system (*let-7C-Gal4; +; tub-Gal80^ts^/UAS-miR-125*). We induced *miR-125* expression either specifically during developmental stages or in adulthood and scored the numbers of CpCs. We found that *miR-125* was capable of increasing the size of the niche only when it was upregulated during pre-adult stages (Fig. 3F, Table S4). Together, these results indicate that the observed *miR-125* GSC niche phenotype is the result of defects that occur during GSC niche formation but not of abnormal somatic cell division or EC *trans-*differentiation in the adult germarium.

### miR-125 coordinates paracrine Notch and endocrine ecdysone signaling in the developing ovary via its target Tom

Next, to test whether Tom also plays a functional role in the establishment of the GSC niche, we analyzed the consequence of *Tom* downregulation using *Tom-RNAi* (Fig. S2D, Table S2) and the *let-7C-Gal4/Gal80^ts^* system. Similar to *miR-125* gain-of-function, downregulation of *Tom* is sufficient to increase the size of the GSC niche only when it is downregulated during pre-adult stages in the *let-7C*-expressing cells, but not during adulthood after stem cell niche establishment is finalized (Fig. 3F, Table S4). This indicates that *miR-125* and its target Tom function in the solitary *let-7C*-expressing TFCs during pre-adult stages to manage the GSC niche assembly.

Importantly, the *miR-125* GSC niche phenotype was similar to the phenotype caused by defective ecdysone signaling (König et al., 2011). Downregulation of ecdysone signaling by the expression of the dominant-negative form of the ecdysone receptor (*hsGal4*; *UAS-EcR^LBD^*) during late larval and early pupal stages results in the increased number of CpCs (Fig. S4B, Table S4). Therefore, we propose a model that the ecdysone-induced *miR-125* downregulates *Tom*, which reprograms the Notch signal-receiving TFC into a Delta-sending cell. According to this model, deregulation of the components downstream of *miR-125* in the *let-7C*-expressing cell should also affect niche formation. To up- or downregulate Tom, Delta or Notch, we used RNAi transgenes driven by *let-7C-Gal4*. Reducing levels of Tom or Delta, as well as overexpression of *miR-125*, Tom or Delta in the *let-7C*-expressing cells lead to an increase in CpC numbers (Fig. S4A-B, Table S4), suggesting that the proper levels of *miR-125*, Tom and Delta are required in the same cell type to establish a proper GSC niche.

### Expression network of the signaling cascade required for the posterior TFC reprograming

As Tom acts as an antagonist of Notch signaling via its interaction with Neur (Bardin and Schweisguth, 2006; De Renzis et al., 2006; Fontana and Posakony, 2009) and is a candidate target for the ecdysone-induced *miR-125*, we hypothesized that the steroid-*miR-125*-Tom-Neur-Delta-Notch signaling cascade controls reprogramming of the posterior TFC from a Notch-active into Notch signal-sending state. To test this hypothesis, we examined the expression network of all of the components of this cascade during CpC specification.

**Fig. 4.**
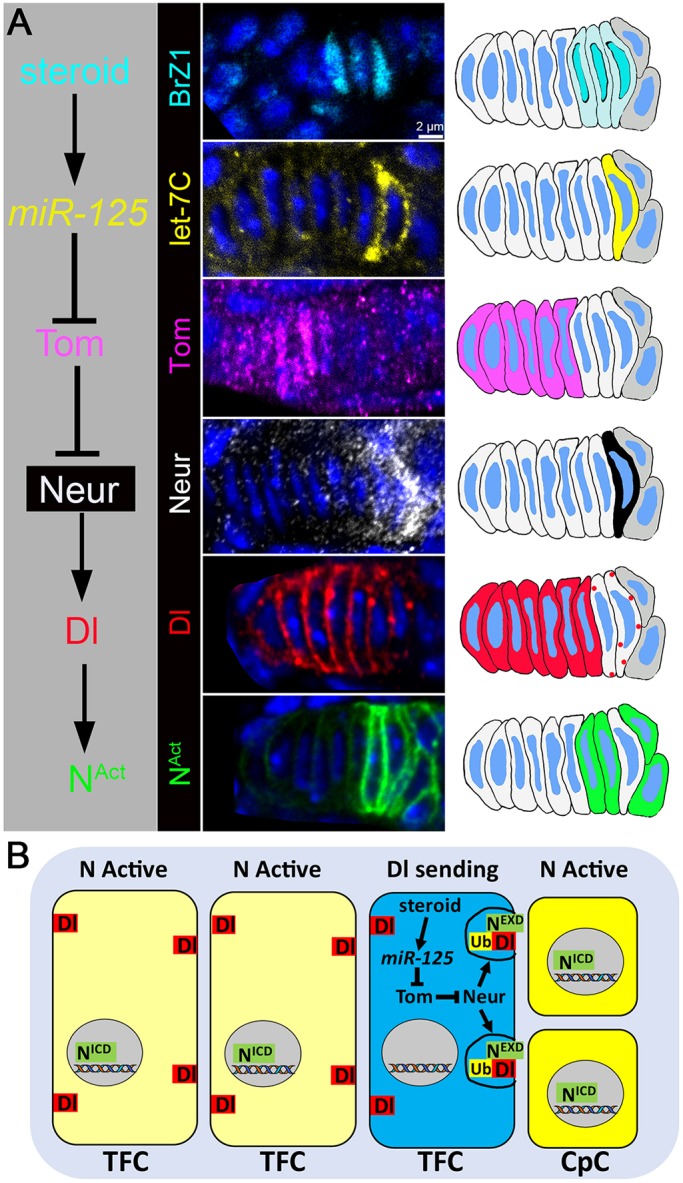
**Steroid-*miR-125*-Tom-Neur-Delta-Notch signaling cascade regulates reprogramming of the anterior TFC.** (A) Confocal images and schematics of expression patterns of components from the steroid-*miR-125*-Tom-Neur-Delta-Notch signaling cascade in TFCs. Ecdysone-induced transcription factor BrZ1 (cyan) and let-7C miRNAs (including *miR-125*, yellow) are expressed in the posterior TFCs adjacent to the germline. The Notch antagonist Tom (magenta) has a reciprocal expression pattern; Tom is detected in the anterior TFCs, as assayed by the expression of Tom-GFP fusion protein, mRNA of which contains *Tom-3′UTR* with *miR-125*-binding site (see Materials and Methods). Ubiquitin ligase Neur (white) is expressed in the posterior TFC that also has Dl present in the vesicles. Neur expression was assayed by the expression of Neur-GFP fusion protein (see Materials and Methods). The anterior TFCs have higher Dl protein levels and no Notch activity, whereas the posterior TFCs adjacent to the germline have reduced Dl levels and are Notch active, except for the most posterior TFC that has vesicular Dl and no Notch activity. (B) Model proposing the role of the steroid-*miR-125*-Tom-Neur-Delta-Notch signaling cascade in the posterior TFC that allows its reprogramming into a Dl-sending state.

As mentioned earlier, we used Broad Z1 as a readout for ecdysone activity (Fig. S2B) and found that it is highly expressed in the early formed TFCs, adjacent to the germline cells (Fig. 4A, BrZ1, cyan). Analysis of the *let-7* complex expression (*let-7C-Gal4>CD8::GFP*) in TFs revealed that miRNAs from the complex, including *miR-125*, are expressed in the posterior TFC located next to the germline (Fig. 4A, *let-7C*, yellow). Whereas *miR-125* was found to be in the posterior TFC, Tom expression (Tom-GFP) had a reciprocal expression pattern and was observed in the anterior TFCs (Fig. 4A, Tom, magenta), suggesting that Tom can be targeted by *miR-125* in TFCs via the expression-tuning mode. Next, we analyzed Neur expression (Neur-GFP) and found that its expression in TFCs was similar to the *let-7C* expression pattern. In particular, it was enriched in the most-posterior TFC (Fig. 4A, Neur, white). Last, we took a closer look at Delta expression and Notch signaling activity in these cells. Although Delta was present in all TFCs, its levels were decreased in the most posterior TFC. Furthermore, the Delta protein appeared to be incorporated into vesicles, suggesting that this cell has activated Delta and acts as a Notch signal-sending cell (Fig. 4A, Delta, red). The switch into a Notch signal-sending state is also supported by the analysis of the Notch activity reporters *E(spl)mß-CD2* and *NRE-EGFP*, expression levels of which were reduced in the most-posterior TFC while still high in other posterior TFCs (Fig. 4A, N^Act^, green and Fig. S1F, arrowhead). In summary, the analyses of the expression patterns and GSC niche phenotypes lead us to propose a model in which, during the larval-to-pupal transition, *miR-125* induced by the ecdysone pulse is expressed in the most posterior TFC. This results in the downregulation of Tom, which de-represses Neur, allowing it to ubiquitylate Delta, which leads to Delta internalization, activation and delivery to the membrane (Fig. 4B). Presence of Delta at the membrane allows it to bind the extracellular domain of the Notch receptor in the six adjacent ISCs, converting them to Notch signal-receiving cells. This promotes their differentiation into CpCs, which concludes GSC niche formation. Importantly, in *miR-125* mutants, the expression of the upstream component of the proposed cascade, BrZ1, was not changed (Fig. S2B), whereas the expression pattern of *miR-125* downstream component, Delta, was altered (Fig. S5). In the posterior TFCs, the membrane Delta levels were not reduced and the Delta-positive vesicle did not appear (Fig. S5C,D), supporting the hypothesis that the presence of *miR-125* positively affects internalization of Delta in a cell-autonomous manner.

### The steroid-miR-125-Notch signaling cascade is required for proper niche establishment

Next, we aimed to verify the proposed model of the miRNA-based coordination of the ecdysone and Notch signaling pathways during the stem cell niche development. If our model (Fig. 4A,B) is correct, then deregulation of any of the signaling cascade components should have an effect on GSC niche formation. Therefore, next we analyzed the architecture of GSC niches in steroid-*miRNA-125*-Tom-Delta-Notch signaling cascade mutants.

**Fig. 5.**
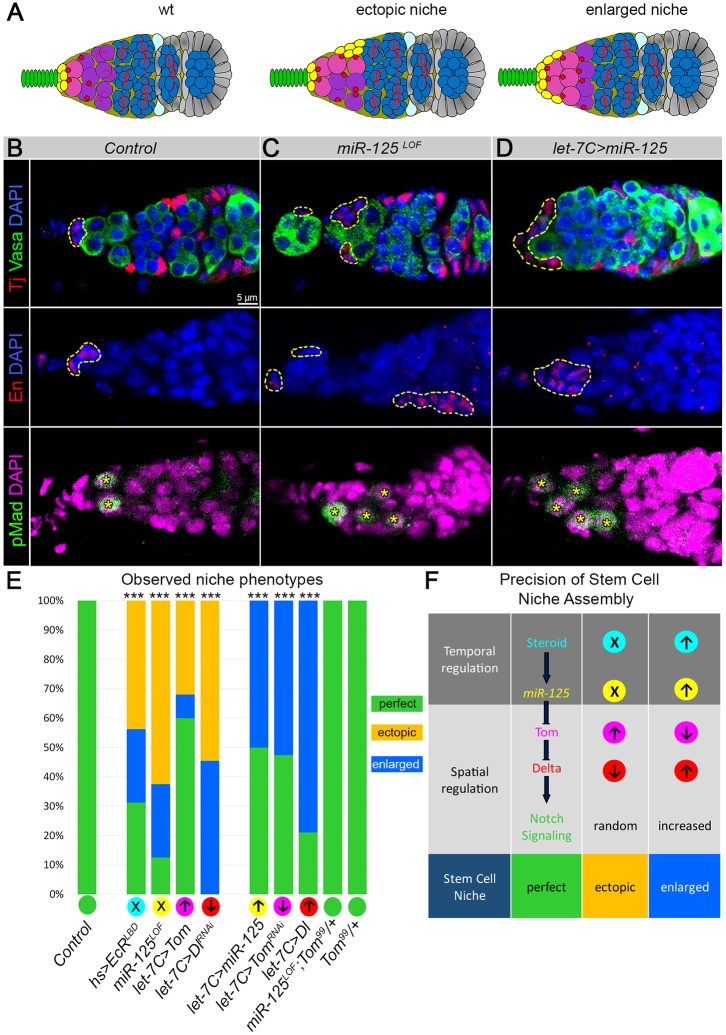
**Steroid-*miR-125*-Tom-Delta-Notch signaling cascade is required for the proper GSC niche establishment.** (A) The adult germaria depicting observed niche phenotypes. TFCs (green), CpCs (yellow), ECs (olive), GSCs (pink), CBs (violet), cyst (blue), follicle stem cells (light blue), spectrosomes (red) and follicle cells (gray). (B) Wild-type germarium shows normal adult GSC niches (outlined by yellow dashed lines) that normally facilitate two or three GSCs (pMad, yellow asterisks). (C) Loss of *miR-125* (*miR-125^LOF^*) leads to the formation of the ectopic niches (dislocated from the anterior tip that host supernumerary GSCs, yellow asterisks). (D) Upregulation of *miR-125* (*let-7C>miR-125*) leads to the enlarged niche phenotype that can host supernumerary GSCs (yellow asterisks). (B-D) CpCs are marked either by Tj or En (red, outlined by yellow dashed lines), GSCs are marked by pMad (green, yellow asterisks) and the germline cells are marked with Vasa (green). (E) Bar graph represents the type and the percentage of the observed niche phenotypes. Two-way tables and χ^2^-test were used to test for statistical significance: ***P≤0.001. (F) Schematics explain the effect of the steroid-*miR-125*-Tom-Delta-Notch signaling cascade deregulation on the stem cell niche assembly. Steroid-induced *miR-125* acts as an intermediary between temporal endocrine steroid and spatial paracrine Notch signaling to convert a Notch-active cell into a Delta-producing cell. This allows Delta ligand to be accurately delivered to the adjacent CpC precursors, so they can acquire Notch-active status, endurance of which is essential for their life-long stem cell niche cell fate. Importantly, this precisely localized delivery of the Delta ligand is crucial to control the precision of the stem cell niche size and architecture. When Delta is not properly activated, it leads to the formation of stem cell niches at ectopic locations. When Delta levels are increased, it causes more niche precursors to adopt the CpC fate, resulting in the enlarged niches.

Interestingly, upon *miR-125* deregulation, higher numbers of CpCs resulted from either the increase in the niche size that was normally positioned at the anterior tip of the germarium and attached to the TF or the appearance of ectopic niches that were detached from the TF and positioned on the side of the germarium (Fig. 5A-D, Fig. S6). In particular, upregulation of *miR-125* using its endogenous promoter (*let-7C>miR-125*) resulted mainly in enlarged niches, whereas *miR-125* deficiency (*miR-125^LOF^*) caused ectopic niches, and this phenotype could be fully rescued by reducing *Tom* by one copy (Fig. 5E, Table S5). Importantly, both ectopic and enlarged niches were fully functional and could host additional GSCs (Fig. 5B-D, Fig. S6, Table S3).

Notably, we observed ectopic niches not only in *miR-125* mutants, but also in the cases of ecdysone signaling deficiency, *Tom* upregulation or *Delta* downregulation in *let-7C*-expressing TFCs (Fig. 5E,F, Fig. S4A, Table S5). Importantly, ectopic niches appeared only in the genotypes that suppress the establishment of the Notch signal-sending TFC. These results can be explained as follows: at the beginning of niche establishment in the larval ovary, all somatic cells (TFCs and ISCs) have the potential to become Notch signal-sending cells. If Notch signal-sending cell fate is not triggered properly in the posterior TFC by the steroid-*miR-125*-Tom-Delta cascade, other undecided cell(s) choose the Notch signal-sending fate randomly at any location, resulting in the formation of ectopic niche(s) (Fig. 5E,F, Table S5). Thus, the bistability of the niche precursors is resolved by activation of the Notch signaling that is induced by the Notch signal-sending TFC, which results in hexagonal patterning and formation of the perfect GSC niche. If this induction does not happen, this bistability is resolved randomly. Importantly, the expression levels of the components of the signaling cascade also have a significant influence on the size of the stem cell niche. Upregulation of Delta in the *let-7C-*expressing TFC leads to formation of enlarged niches in which CpCs are correctly positioned at the TF base; however, their numbers are increased in response to high Delta levels. The same effect has downregulation of *Tom* or upregulation of ecdysone signaling its dependent miRNA: *miR-125* (Fig. 5E,F, Table S5).

In summary, these data indicate that CpCs can be specified either via lateral inhibition, in which a CpC precursor acquires Notch signal-sending status randomly due to transcriptional noise, or via peripheral induction, whereby Delta is produced by a reprogrammed TF cell. The latter process is controlled via steroid-induced *miR-125*, which targets a negative regulator of Notch signaling: Tom. Without proper communication between the components of the steroid-*miR-125*-Tom-Delta-Notch signaling cascade, TFC reprogramming does not occur, which leads to formation of stem cell niches at ectopic locations. Precise expression of *miR-125* ensures reprogramming of the Notch-active posterior TFC into a Notch signal-sending cell, which via peripheral induction activates Notch signaling in the adjacent CpCs, resulting in hexagonal tessellation and formation of an accurate GSC niche consisting of six CpCs. Thus, the spatiotemporal interaction between Notch and steroid signaling resolves the niche precursor cell bistability, which is extrapolated onto the patterning of the adult stem cell niche.

## DISCUSSION

Here, we have investigated the roles of the Notch and steroid hormone signaling pathways during development of the *Drosophila* ovary and found that both pathways specify the order and accuracy of stem cell niche assembly in this organ. We found that each TF serves as a source of the Delta ligand, which induces Notch signaling in the adjacent niche cell precursors. However, during their differentiation, posterior TFCs acquire Notch signal-receiving status. Therefore, to determine an accurate niche cell pattern, at least one TFC has to change its active Notch signaling status and become a Delta-sending cell. Now, we have identified that a single miRNA, *miR-125*, is necessary to shift the balance between a Notch signal-receiving to a Notch signal-sending cell fate. To do so, steroid-induced *miR-125* is expressed in the TFCs proximal to the germline, where it targets a Notch signaling antagonist: Tom. Downregulation of Tom supports post-transcriptional Delta processing and activation, promoting a Delta-sending cell fate. Activated Delta induces Notch signaling via hexagonal tiling in the neighboring niche cell precursors and converts them to CpCs, which serve as a lifelong niche for the germline stem cells. This newly identified steroid-*miR-125*-Tom-Delta-Notch signaling cascade explains how a cell that was previously Notch active changes its fate to a Notch signal-sending status to achieve the maximum precision during the formation of the germline stem cell niche. Although the importance of the Notch signaling status maintenance has been well documented in multiple cell types in many organisms, it has not been reported in any system that Notch signaling status can be changed. These data for the first time show that a Notch-signal receiving cell can change its fate and be reprogrammed into a Delta-sending cell. As Notch signaling is involved in virtually all biological processes, it is important to recognize that the cellular status regarding Notch activity can be changed. Intriguingly, Notch signaling has been shown to play an essential role in the assembly of all well-characterized stem cell niches in *Drosophila* ovarian, testicular and intestinal (Mathur et al., 2010; Okegbe and DiNardo, 2011; Song et al., 2007; Ward et al., 2006). Similarly, in mammals, Notch signaling is active in one of the best studied niches: the bone marrow hematopoietic stem cell niche (Kushwah et al., 2014; Maillard, 2014; Weber and Calvi, 2010). In addition, maintenance of multiple adult stem cell types in many organisms also depends on Notch signaling (Ables et al., 2011; Carlson et al., 2009; Maillard, 2014), suggesting that Notch signaling has a conserved role in the stem cell niches.

The Notch activity can deteriorate or be lost upon ageing or starvation in niche cells, and this process could be reversible (Bonfini et al., 2015; Tseng et al., 2014). In addition, it has been shown that a cell can have an intrinsic miRNA-driven mechanism to regulate Notch activity (Bonev et al., 2012). In particular, short-period oscillations of the Notch effector Hes1 are necessary to maintain the self-renewing characteristics of neural progenitors. This process is controlled by *miR-9*, which targets Hes1 and at the same time, its expression is controlled by Hes1. This generates a double negative-feedback loop and results in out-of-phase oscillations. Owing to the differences in mature miRNA and Hes1 protein stabilities, over time, *miR-9* levels increase and Hes1 oscillations dampen, leading to neuronal differentiation (Bonev et al., 2012). Therefore, it would be interesting to study whether, in the ovarian stem cell niche precursors, Delta expression and Notch activation oscillate before becoming locally confined via the steroid-*miR-125*-Tom pathway.

The steroid-dependent miRNA *miR-125* is produced from the polycistronic *let-7* complex gene region, which encodes two additional miRNAs: *let-7* and *miR-100*. Interestingly, it has been found that co-transcribed miRNAs can function independently during different developmental stages and have non-overlapping functions even when they have a common target (Chawla et al., 2016). Previously, *let-7* has been shown to be involved in *Drosophila* gametogenesis, whereas the role for *miR-125* in the ovaries has not been studied before. Interestingly, *let-7* is required for the maintenance of sexual identity of testicular and ovarian somatic cells, and for the communication between the soma and germline (Fagegaltier et al., 2014; König and Shcherbata, 2015). It fine-tunes the cell adhesion that affects Wg/Wnt signaling strength, adjusting the germline stem cell progeny chromatin status; thus, coordinating the speed of their differentiation with organismal needs. Previous studies showed that *let-7* acts to target a negative regulator of ecdysone signaling: the transcription factor Abrupt (Caygill and Johnston, 2008; König and Shcherbata, 2015; Kucherenko et al., 2012). Abrupt is a very potent regulator of epithelial and neuronal cell identity (Grieder et al., 2007; Kucherenko et al., 2012; Turkel et al., 2013). By targeting such an important cell fate determinant that also is a negative regulator of steroid signaling that induces the expression of miRNA per se, *let-7* confers steroid signaling robustness via a positive-feedback loop.

Here, we have found that *miR-125* acts in developing ovary to destabilize a positive-feedback loop established by Notch signaling. Perturbation of *miR-125* expression results in the supernumerary niche cell appearance, a phenotype that is similar to one observed upon perturbation of Notch signaling. We have identified Tom (Twin of M4 or Bardu) as a relevant *miR-125* target, deregulation of which also causes the enlarged niches. Tom overexpression has been shown to block Neur-dependent Notch signaling (Bardin and Schweisguth, 2006). Interestingly, genes from the Bearded family evolved as the result of gene duplication (Chanet et al., 2009); however, only *Tom* contains a unique 3′UTR that has a predicted *miR-125* target site. Tom acts as an antagonist of Notch-active cell fate via repression of Delta and, at the same time, Tom expression is positively regulated by Notch signaling (Chanet et al., 2009; Lai et al., 2000; Zaffran and Frasch, 2000). Therefore, it functions in a positive-feedback loop to reinforce the maintenance of the Notch signal-receiving cell fate, which is important for terminal differentiation and safeguarding of the TFC fate. However, for the establishment of the proper stem cell niche, this positive reinforcement of Notch signaling has to be interrupted, because in order to induce the stem cell niche in the accurate location, TF should become the source of Delta.

Thus, *miR-125* expression allows the positive-feedback loop of Notch activation to be broken and enables the conversion of a cell from Notch signal receiving into Notch signal sending. Activation of a Notch signal-sending fate suppresses a similar fate and leads to activation of a Notch signal-receiving fate in the neighboring niche cell precursors via peripheral induction. This serves as an additional security mechanism to disallow formation of ectopic niches by mistake, either by random suppression or promotion of Notch signal-receiving or signal-sending cell state in the bistable ISCs that express both the Delta ligand and Notch receptor. Therefore, based on our data, we believe that the accurate assembly of the stem cell niche is accomplished by spatiotemporal coordination of Notch and steroid signaling by *miR-125*. Any discord in this regulation leads to formation of abnormal stem cell niches. These abnormal niches could be small, enlarged or ectopic, resembling a little hut, a mansion or several separate domiciles where the stem cells station. Because the stem cell number is positively correlated with the stem cell niche size, securing the stereotypical niche architecture has a significant impact on tissue homeostasis, tumorigenesis, fitness and reproduction.

## MATERIALS AND METHODS

### Fly strains and genetics

Fly stocks were maintained at 25°C on a standard cornmeal-agar diet in the controlled environment (constant humidity and light-dark cycle) unless otherwise stated. As a control, either *let-7C^GK1^* crossed to *w^1118^* or *OregonR* crossed to *w^1118^* lines were used.

To generate flies mutant for *miR-125*, referred to as *miR-125^LOF^*, two mutant lines *let-7C^GK1^/CyO-GFP*; let-7C^ΔmiR-125^ and *let-7C^KO1^/CyO-GFP*; let-7C^ΔmiR-125^ were used to obtain final genotype *let-7C^GK1^*/*let-7C^K01^*; let-7C^ΔmiR-125^. *let-7C^GK1^/CyO-GFP* and *let-7C^KO1^/CyO-GFP* lack the whole sequence encoding the *let-7* complex (*let-7C*) miRNAs (Sokol et al., 2008). let-7C^ΔmiR-125^ is an insertion of the genomic locus on the 3rd chromosome that restores the expression of all *let-7C* miRNAs, except for *miR-125*. In addition, *let-7C^GK1^/CyO* line contains the transcriptional activator *Gal4* under control of the *let-7*C promoter and, therefore, was used as the *let-7C* endogenous driver line. To induce *Gal4* expression during developmental stages, *tub-Gal80^ts^* was introduced to a *let-7C^GK1^*/*CyO* line, referred to as *let-7C^ts^*. To upregulate *miR-125* levels, *UASt-miR-125* (Caygill and Johnston, 2008) was used. To manipulate Notch signaling, *UASt-N^CA^* and *UASp-Dl* (gifts from Hannele Ruohola-Baker, University of Washington, Seattle, USA), *UASt-Dl-RNAi* [Bloomington Drosophila Stock Center (BDSC), 28032], *UASt-Dl-RNAi* (BDCS, 36784), *UASt-Dl* (BDSC, 26695), *UASt-N^RNAi^* (BDSC, 27988) lines were used. To downregulate Notch levels *N^ts1^* mutants were kept at semi-restrictive temperature, 25°C, through all stages of development. For *Tom* overexpression or downregulation, *UASt-Tom* (a gift from Manfred Frasch, Friedrich-Alexander-University, Erlangen-Nürnberg, Germany) and *UAS-Tom-RNAi* [Vienna Drosophila RNAi Center (VDRC), 36613] lines were used, respectively. To downregulate ecdysone signaling, the *hs-EcR-LBD* line (BDSC, 23656) was heat shocked for 25 min at 37°C at EL3 stage (72 h AEL) and dissected as prepupa (120 h AEL). For ectopic expression, the following driver lines were used: *nos-Gal4*, *bab1-Gal4/TM6*, *ptc-Gal4*, *sca-Gal4* (BDSC) and *let-7-Gal4*. To monitor Tom and Neur expression, *Tom-GFP* (YB0063) and *Neur-GFP* (YD1143) lines from FlyTrap were used. These GFP insertions are made by insertion of P-element-containing artificial exon encoding GFP flanked by splice acceptor (SA) and splice donor (SD) sequences so that expression of GFP relies on splicing into mature mRNAs and in-frame fusion and contains endogenous 3′UTR (Quinones-Coello et al., 2007). To monitor Delta expression, the *Dl-GFP* MiMiC line (BDSC, 37855) was used. To generate the rescue line, *Tom^99^* mutant allele was introduced to the *let-7C^K01^*/CyO-GFP; let-7C^ΔmiR-125^ line and crossed to *let-7C^GK1^*/CyO-GFP; let-7C^ΔmiR-125^ to obtain the final genotype: *let-7C^GK1^*/*let-7C^K01^*; let-7C^ΔmiR-125^/Tom^99^. To monitor Notch activity, the following reporter lines were used: a Notch signaling reporter in which the sequence encoding rat CD2 protein is inserted downstream of the Enhancer of Split [E(spl)mβ] promoter [*E(spl)mß-CD2*] that is activated by Notch signaling (de Celis et al., 1998); and a NRE-GFP reporter that expresses EGFP under the control of Notch-responsive element (NRE, BDSC 30728). To mark the ECM in ovaries, *LanA::GFP* line (VDRC, 318155) was used.

### Immunohistochemistry

Immunofluorescent staining was carried out using a standard procedure (König and Shcherbata, 2013). The following primary antibodies were used: mouse anti-En (1:20), mouse anti-β-Gal (1:20), mouse anti-BrZ1 (1:20), mouse anti-N^ICD^ (1:20) and mouse anti-Dl (1:20), all from Developmental Studies Hybridoma Bank; rabbit anti-phosphorylated Mad (pMad, 1:5000; a gift from Ed Laufer, Columbia University, New York, USA), rabbit anti-Vasa (1:5000; a gift from Herbert Jäckle, Max Planck Institute for Biophysical Chemistry, Goettingen, Germany), mouse anti-CD2 (1:100, BioLegend, 100107), guinea pig anti-Traffic Jam (Tj, 1:5000; a gift from D. Godt, University of Toronto, Canada) and chicken anti-GFP (1:5000, Abcam, ab13970). Secondary antibodies were: goat anti-rabbit Alexa 488, goat anti-chicken Alexa 488 and goat anti-guinea pig Alexa 647 (1:500, Life Technologies, A-11034, A-11039, A-21450); goat anti-mouse Cy3 IgG1 and goat anti-mouse IgG2a Alexa 488 (1:250, Jackson ImmunoResearch Laboratory). For visualizing cell nuclei, DAPI dye was used (Sigma). Samples were analyzed using a confocal microscope (Zeiss LSM 700). For making figures, Adobe Photoshop software was used.

### Analyses of the GSC niche cell numbers and morphology

Cells that express both En and Traffic Jam (Tj), and have the lentil-shape morphology were identified as CpCs. To analyze the number of CpCs and GSCs per germarium, *z*-stack confocal images of the germarium of adult ovaries with 1 μm intervals were captured. CpCs were identified by En staining and a lentil shape. Germline cells positive for pMad staining were identified as GSCs. Comparison between the numbers of CpCs and GSCs per germarium among different samples were carried out using one-way ANOVA with post-hoc Tukey HSD test. For all the experiments, at least two independent biological replicates were carried out.

### Luciferase reporter assay

To generate the *Tom-3′UTR* sensor, a 263 bp region containing putative *miR-125* binding site was amplified from genomic *Drosophila melanogaster* DNA by polymerase chain reaction with primers that included enzymatic digestion sites for *Not*I and *Xho*I as follows: forward, TACGTGCGGCCGCGCCTAAACATCGCCAGGATGC; reverse, CCACCATGGCTCGAGCGATAGTAACGCTTGATTGTG (the additional bases for enzyme cut sites are underlined). The fragment was subsequently cloned into *Not*I and *Xho*I restriction sites downstream of *Renilla* luciferase gene in the psiCHECK-2 vector (Promega). *Drosophila* S2R+ cells (DGRC Indiana) were seeded to 8×10^4^ in a 96-well cell culture plate one day after splitting 1:6. Cells were transfected using Effectene transfection reagent (Qiagen) with the following amounts: 50 ng of empty psiCHECK-2 (Promega), 50 ng of *psiCHECK-2* with the *Tom-3′UTR* sensor, 25 ng of *act-Gal4* and 50 ng of *pUASt-miR-125* plasmid (kindly provided by Eric Lai, Memorial Sloan Kettering Cancer Center, New York, USA). Approximately 72 h after transfection, the cells were subjected to the Dual-Glo luciferase assay (Promega). Both *Firefly* (control reporter) and *Renilla* luciferase (altered *3′-UTR* experimental reporter) levels were measured to achieve optimal and consistent results. Plates were analyzed on a Wallac 1420 luminometer. Non-transfected cells were used for blank subtraction from the raw luminescence counts and control reporter counts (*Firefly* luciferase) were normalized to experimental reporter (*Renilla* luciferase) counts to determine the fold repression of *Renilla* luciferase activity. The determined *Renilla* luciferase activity in the presence of the empty *psiCHECK-2* plasmid was subtracted from that in the presence of the *psiCHECK-2-Tom-3′UTR* plasmid without the presence of transfected microRNA plasmids. Then the *Renilla* luciferase activity was determined with the empty *psiCHECK-2* and *psiCHECK-2-Tom-3′UTR* plasmid in the presence of the *miR-125*, and the difference between these values was calculated. The difference in luminescence between *psiCHECK-2* plasmid and *psiCHECK-2-Tom-3′UTR* plasmid in the presence of *miR-125* was then normalized to the difference between *psiCHECK-2* plasmid and *psiCHECK-2-Tom-3′UTR* plasmid without *miR-125* to determine the fold reduction caused by the presence of the endogenous *miR-125*. All transfections were carried out in triplicate to determine the average and standard deviations, and two-tailed Student's *t*-test was used to test for statistical significance.

### RNA preparation and real-time quantitative PCR (RT-qPCR)

Total RNA was extracted using Trizol reagent (Invitrogen) following the manufacturer's protocol and treated with DNase1 (Invitrogen). To detect miRNA levels, TaqMan microRNA assays (Applied Biosystems) were used to reverse transcribe from RNA and amplify cDNA specific to *let-7*, *miR-100*, *miR-125* and *2S rRNA* as an endogenous control on a StepOne Plus thermocycler (Applied Biosystems). qPCR was carried out using the Taqman qPCR master mix (Applied Biosystems). For the determination of mRNA transcript levels, RNA was converted into cDNA using the High Capacity reverse transcription kit (Applied Biosystems) following the manufacturer's directions. To detect *Tom mRNA* the forward and reverse primers TGGCTTCAATTGAGCGAGCT and TCACGCAGTTAGTTATTCTC, respectively were used. As an endogenous control for qPCR reactions, Ribosomal Protein L32 (RpL32) with the following forward and reverse primers CCAGGATGCACAGTTCCACT and GAACATGCGAAGCTGAACCC, respectively, were used. All reactions were run at least in triplicate with appropriate blank controls. The threshold cycle (C_T_) was defined as the fractional cycle number at which the fluorescence passes a fixed threshold. The ΔC_T_ value was determined by subtracting the average *RpL32* or *2S rRNA* C_T_ value from the average tested C_T_ value of target mRNA or miRNA, correspondingly. The ΔΔC_T_ value was calculated by subtracting the ΔC_T_ of the control sample from the ΔC_T_ of the experimental sample. The relative amounts of miRNAs or target mRNA is then determined using the expression 2^−ΔΔCT^.

### Target prediction

For the prediction of targets of the *let-7C* miRNAs, Target Scan 6.2 (Kheradpour et al., 2007), miRNA Target Gene Predictions at EMBL (Stark et al., 2003) and miRANDA (microRNA.org) (Enright et al., 2003) databases were used. To obtain the list of Notch signaling pathways genes, the FlyBase gene ontology database was used (flybase.org).

## Supplementary Material

Supplementary information

Supplementary information
