# Supplementary material for: Stereotypical architecture of the stem cell niche is spatiotemporally established by miR-125-dependent coordination of Notch and steroid signaling
Source: Development. 2018 Feb 1;145(3):dev159178. doi: 10.1242/dev.159178 (PMC5818007; doi:10.1242/dev.159178)
Supplement: Supplementary information [file develop-145-159178-s1.pdf]

Supplementary Information

Supplementary Tables

Table S1. *miR-125* targets *Tom-3`UTR*

miRNA expression plasmid	Renilla /Firefly luciferase ratio (*psiCHECK-2*)	Renilla /Firefly luciferase ratio (*psiCHECK-2-Tom- 3'UTR*)	Relative Luciferase levels (*psiCHECK-2-Tom-3'UTR /psiCHECK-2*)	Relative Luciferase levels [a]
[a] none	0.17 ± 0.02	0.24 ± 0.03	1.41 ± 0.18	1.00 ± 0.17
miR-125	0.96 ± 0.44	0.35 ± 0.01	0.36 ± 0.01	0.26 ± 0.02<br>P=0.0042

[a] Values in the presence of miRNA expressing plasmids were normalized to the downregulation that occurred in the cells with no miRNA-expressing plasmid present.
Firefly luciferase expression is used as an endogenous control where *Tom 3'UTR* was cloned into Renilla luciferase gene. AVE±STDEV values are reported. Two-tailed Student's t-test was used to test for statistical significance.

Development • Supplementary information

Table S2. Expression levels of *miR-125, let-7* and *miR-100* in controls and *miR-125^{LOF}* mutants and the efficiency of *Tom* downregulation by *Tom-RNAi*

Genotype	***miR-125*** **(C_T)**	***2S*** **(C_T)**	**[a] ΔC_T**	**[b] $\Delta\Delta C_T$**	**[c] Relative *miRNA* level**
Control (*Oregon x w^{1118}*)	20.51±0.06	8.15±0.10	12.36±0.06	0.00±0.06	1.00±0.04
miR-125^{LOF}	35.94±0.05	7.87±0.12	28.07±0.04	15.71±0.04	0.00±0.00 P=2.4E-6
Genotype	***let-7*** **(C_T)**	***2S*** **(C_T)**	**Δ C_T**	**$\Delta\Delta C_T$**	**Relative *miRNA* level**
Control (*Oregon x w^{1118}*)	23.47±0.18	8.15±0.10	15.32±0.18	0.00±0.18	1.01±0.13
miR-125^{LOF}	23.38±0.22	7.87±0.12	15.51±0.22	0.18±0.22	0.88±0.13 P=0.311
Genotype	***miR-100*** **(C_T)**	***2S*** **(C_T)**	**ΔC_T**	**$\Delta\Delta C_T$**	**Relative *miRNA* level**
Control (*Oregon x w^{1118}*)	21.84±0.11	8.15±0.10	13.69±0.11	0.00±0.11	1.00±0.08
miR-125^{LOF}	21.76±0.09	7.87±0.12	13.90±0.08	0.20±0.06	0.87±0.05 P=0.073
Genotype	***Tom*** **(C_T)**	***Rpl32*** **(C_T)**	**ΔC_T**	**$\Delta\Delta C_T$**	**Relative *mRNA* level**
Control (*Oregon x w^{1118}*)	21.28±1.94	15.26±0.08	6.01±3.35	0.00±0.06	1.00±0.04
Act>Tom^{RNAi} (*Actin-Gal4/UASt - Tom^{RNAi}*)	22.84±1.15	15.01±0.05	6.80±2.09	1.13±0.25	0.46±0.08 P=6.1E-6

[a] - the ΔC_T value is determined by subtracting the average C_T value of endogenous control gene (2S for miRNAs or Rpl32 for mRNA) from the average miRNA or mRNA C_T value.

[b] - the calculation of $\Delta\Delta C_T$ involves subtraction by the ΔC_T calibrator value (ΔC_T value in *OregonR/w^{1118}*).

[c] - the range is given for relative levels determined by evaluating the expression: $2^{-\Delta\Delta CT}$.

AVE±STDEV values are reported from experiments done in triplicates. Two-tailed Student's t-test was used to test for statistical significance

Development • Supplementary information

Table S3. Deregulation of *miR-125* results in the increased GSC niches that host supernumerary GSCs

Genotype	Number of CpCs AVE±STDEV	Number of germaria analyzed	Number of GSCs AVE±STDEV	Number of germaria analyzed
Control (*let-7C^{GK1} x w^{1118}*)	6.3±0.6	16	2.5±0.5	11
miR-125^{LOF} (*let-7C^{GK1}/let-7C^{K01}; P{W8, let-7C$^{\Delta miR\text{-}125}$*)	8.5±2.3 P=0.001	44	3.4±1.1 P=0.001	28
Rescue (*let-7C^{GK1}/let-7C^{K01}; P{W8, let-7C$^{\Delta miR\text{-}125}$/Tom^{99}*)	6.2±0.6 P=0.899	20	2.6±0.8 P=0.4790	20
Tom^{99}/+	6.1±0.4 P=0.085	24	2.3±0.8 P=0.794	27
ptc>miR-125 (*UAS-miR-125/ptc-Gal4*)	5.4±0.7 P=0.794	21	2.6±0.5 P=0.900	20
bab1>miR-125 (*UAS-miR-125/bab1-Gal4*)	7.3±1.1 P=0.001	20	4.1±0.7 P=0.001	21
let-7C>miR-125 (*let-7C^{GK1}/+;UAS-miR-125/+*)	7.7±0.9 P=0.014	31	3.7±1.0 P=0.001	12

One-way ANOVA with post-hoc Tukey HSD was used to test for statistical significance

Development • Supplementary information

Table S4. Deregulation of the steroid-*miR-125*-Tom-Delta-Notch signaling cascade affects GSC niche development

Genotype	**Conditions**	**Number of CpCs AVE±AVEDEV**	**Number of germaria analyzed**
Control (*let-7C*^{GK1} *x w*^{1118})	18° C	6.0±0.9	22
let-7C^{ts}*>miR-125* (*let-7C*^{GK1}/+; *UAS-miR-125/ tub-Gal80*^{ts})		6.0±0.9 P=0.900	10
let-7C>Tom^{RNAi} (*let-7C*^{GK1}/+; *UAS- Tom*^{RNAi}/ *tub-Gal80*^{ts})		6.1±1.2 P=0.900	16
Control (*let-7C*^{GKts1} *x w*^{1118})	shifted to 29°C as adults	5.9±1.2 P=0.900	17
let-7C^{ts}*>miR-125* (*let-7C*^{GK1}/+; *UAS-miR-125/tub-Gal80*^{ts})		6.4±1.0 P=0.900	17
let-7C>Tom^{RNAi} (*let-7C*^{GK1}/+; *UAS- Tom*^{RNAi}/ *tub-Gal80*^{ts})		5.8±1.3 P=0.900	18
Control (*let-7C*^{GK1} *x w*^{1118})	29° C	5.8±1.0 P=0.900	18
let-7C^{ts}*>miR-125* (*let-7C*^{GK1}/+; *UAS-miR-125/tub-Gal80*^{ts})		7.8±0.9 P=0.002	14
let-7C>Tom^{RNAi} (*let-7C*^{GK1}/+; *UAS- Tom*^{RNAi}/ *tub-Gal80*^{ts})		7.3±0.9 P=0.027	16
Control (*let-7C*^{GK1} *x w*^{1118})	25° C	5.5±0.5	12
let-7C>Tom^{RNAi} (*let-7C*^{GK1}/+; *UAS-Tom*^{RNAi}/+)		7.1±0.9 P=0.006	10
let-7C>Tom (*let-7C*^{GK1}/+; *UAS-Tom/+*)		8.2±1.1 P=0.001	18
let-7C>N^{CA} (*let-7C*^{GK1}/+; *UAS-Notch*^{CA}/+)		7.0±0.9 P=0.007	12
let-7C>N^{RNAi} (*let-7C*^{GK1}/+; *UAS-Notch*^{RNAi}/+)		6.9±0.7 P=0.005	16
let-7C>Dl (*let-7C*^{GK1}/+; *UAS-Dl/+*)		7.3±0.8 P=0.001	17
let-7C>Dl^{RNAi} (*let-7C*^{GK1}/+; *UAS-Dl*^{RNAi}/+)		8.2±1.0 P=0.001	11
EcR^{LBD} (*hs-Gal4/UAS-EcR*^{LBD}) heat shocked for 25' at 37°C at EL3 stage (72h AEL)		8.2±1.5 P=0.001	18

One-way ANOVA with post-hoc Tukey HSD was used to test for statistical significance. All samples were compared to the controls at the same conditions

Table S5. Steroid-*miR-125-Tom-Delta-Notch* signaling cascade is important for proper GSC niche architecture

Genotype	Observed niche phenotypes			P-value	Number of germaria analyzed
	Perfect	Enlarged	Ectopic		
Control (*let-7C^{GK1} x w^{1118}*)	100%	0%	0%	-	38
EcR^{LBD} (*hs-Gal4/UAS-EcR^{LBD}*) heat shocked for 25 min at 37°C at EL3 stage (72h AEL)	31%	25%	44%	P=7.5E-08	16
let-7C>miR-125 (*let-7C^{GK1}/+;UAS-miR-125/+*)	50%	50%	0%	P=5.7E-06	28
miR-125^{LOF} (*let-7C^{GK1}/let-7C^{K01}; P{W8, let-7C$^{\Delta miR\text{-}125}$}*)	13%	25%	63%	P=1.2E-11	24
let-7C>Dl (*let-7C^{GK1}/+; UAS-Dl/+*)	21%	79%	0%	P=1.4E-09	19
let-7C>Delta-RNAi (*let-7C^{GK1}/+; UAS-Dl^{RNAi}/+*)	0%	45%	55%	P=1.1E-30	11
let-7C>Tom (*let-7C^{GK1}/+; UAS-Tom/+*)	60%	8%	32%	P=3.1E-12	25
let-7C>Tom^{RNAi} (*let-7C^{GK1}/+; UAS-Tom^{RNAi}/+*)	47%	53%	0%	P=7.9E-08	19
Rescue (*let-7C^{GK1}/let-7C^{K01}; P{W8, let-7C$^{\Delta miR\text{-}125}$}/Tom^{99}*)	100%	0%	0%	P=1	13
Tom^{99}/+	100%	0%	0%	P=1	24

Two-way tables and χ^2-test were used to test for statistical significance

Supplementary Figures

Supplementary Figure 1

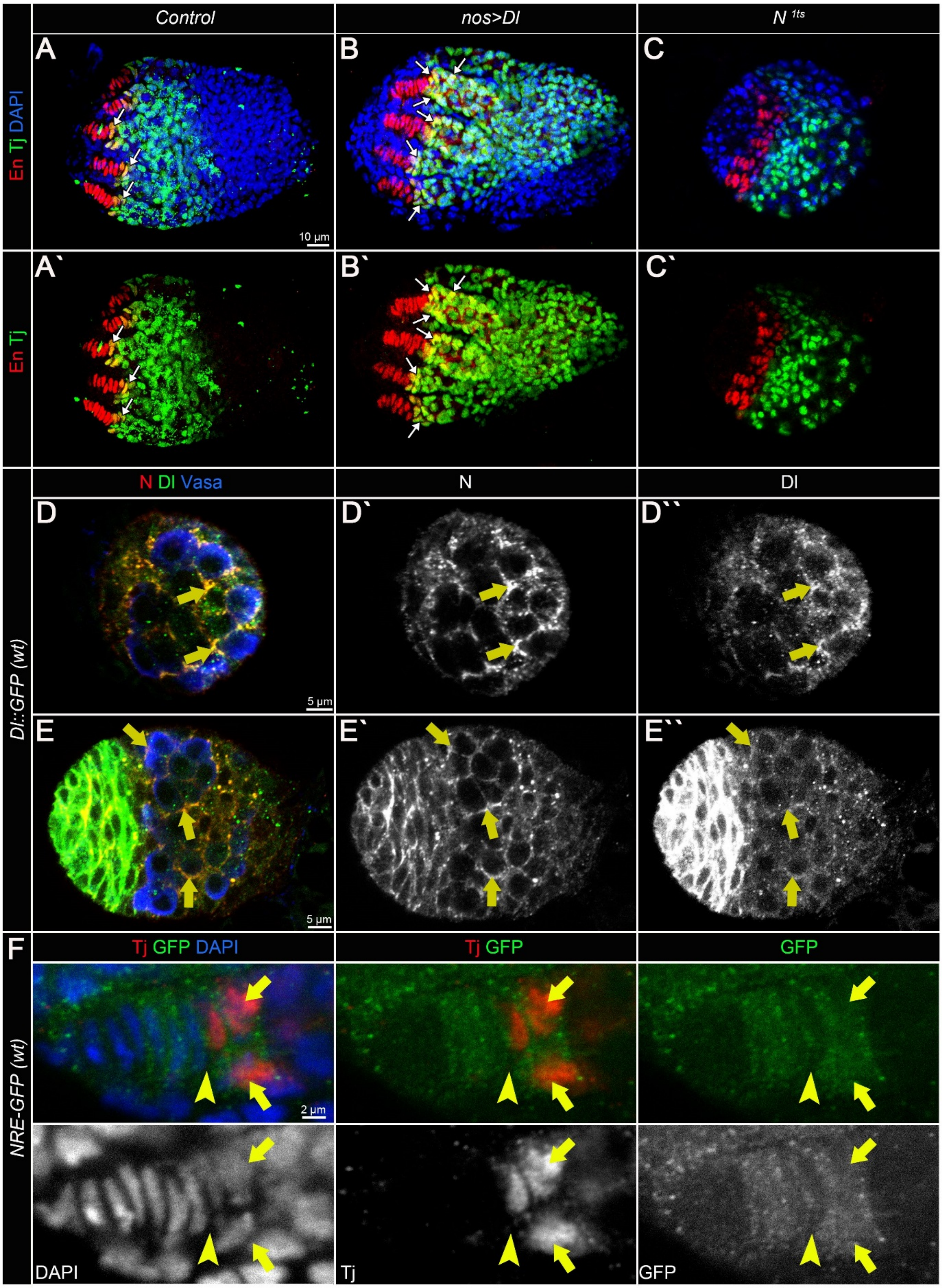

Development • Supplementary information

Figure S1. The strength of Notch signaling controls GSC niche formation

A-C. Ectopic activation of Delta leads to the formation of additional CpCs (arrows, E), while upon Notch downregulation (N^{lts}), very few CpCs can be observed (arrows, F) when compared to *Control* (D). TFCs are marked by En (red), CpCs are marked by En+Tj (yellow, arrows) and ECs are marked by Tj (green).

D-E. Expression pattern of the Notch ligand, Delta (Dl::GFP) and the Notch receptor (anti-Notch intracellular domain antibodies) in the developing ovary at the second instar larva (L2, D) and early third instar larva (EL3, E) stages. Note that ISCs, which are the somatic cells intermingled with the germline cells (Vasa, blue) co-express Notch and Delta (olive arrows) and are "undecided" with regards to their Notch signaling status.

F. Expression pattern of the Notch activity reporter NRE-EGFP in the developing GSC niche unit at the prepupal stage. Note that the posterior TFC (arrowhead) adjacent to the forming CpCs (arrows) shows the reduced Notch activity.

Supplementary Figure 2

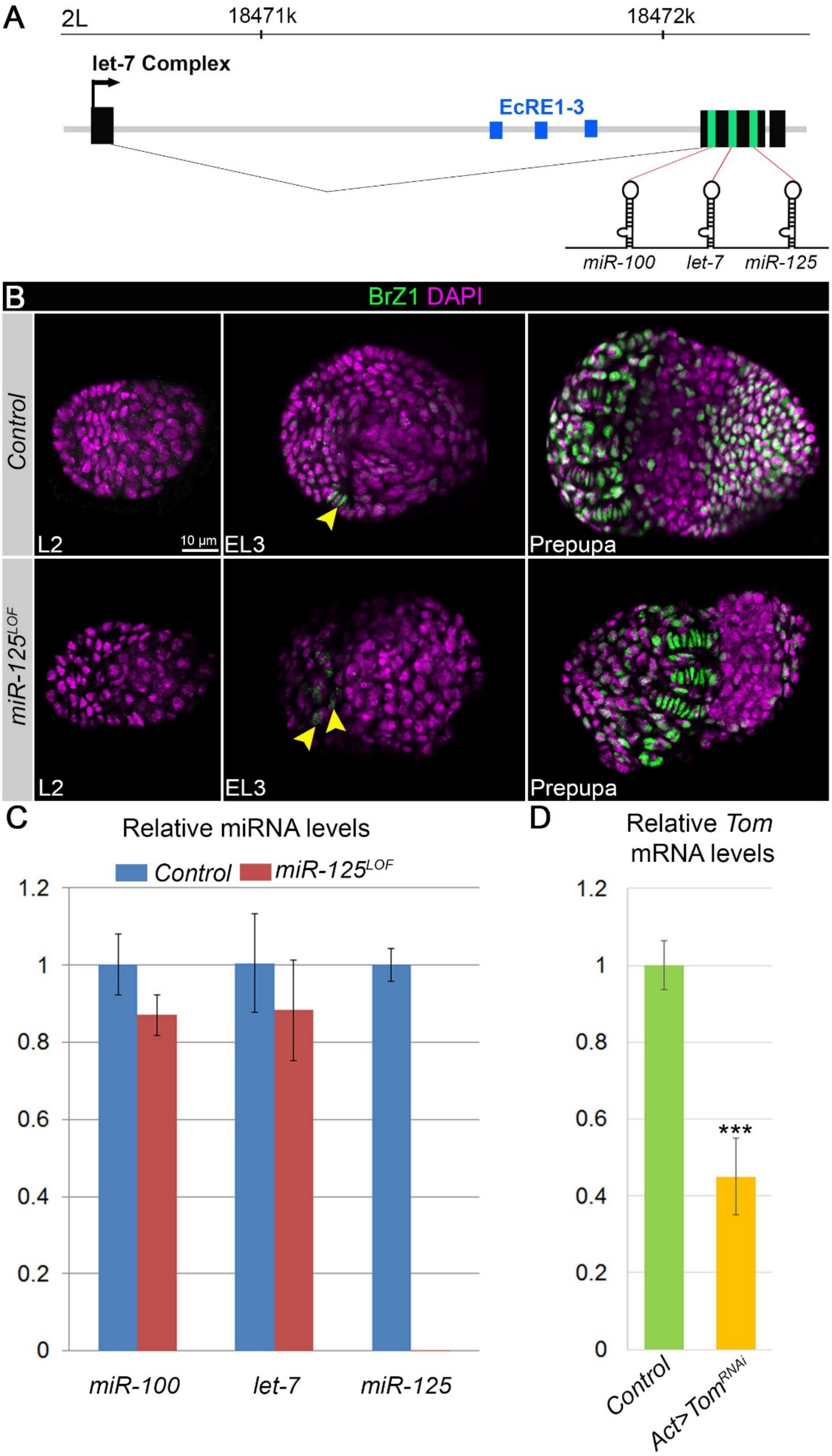

Development • Supplementary information

Figure S2. *let-7* complex miRNA are expressed in the developing ovary

A. Structure of the *let-7* complex gene region [modified from (Chawla and Sokol, 2012)] with the ecdysone response elements (EcRE, blue).

B. Expression of the ecdysone-activated transcription factor BrZ1 (green) during different stages of ovary development. BrZ1 is first detected in the newly formed TFCs at EL3 stage; afterwards, it is broadly expressed in the somatic cells, including TFCs. BrZ1 expression pattern is not altered in *miR-125* mutants.

C. Bar graph represents relative levels of miRNAs from the *let-7C* (*miR-100*, *let-7* and *miR-125*), in controls and *miR-125* mutants (*let-7-C*^{K01}/*CyO-GFP; let-7-C*$^{\Delta miR\text{-}125}$).

AVE±STDEV values are reported from the experiments done in triplicates.

D. *Tom* mRNA levels in *Control* and upon ubiquitous downregulation of *Tom* (*Act-Gal4/UAS-Tom*^{RNAi}). Bar graph represents relative levels *Tom* mRNA.

AVE±STDEV values are reported from two biological experiments done in triplicates. Two-tailed Student's t-test was used to test for statistical significance: ***P≤0.001.

Development • Supplementary information

Supplementary Figure 3

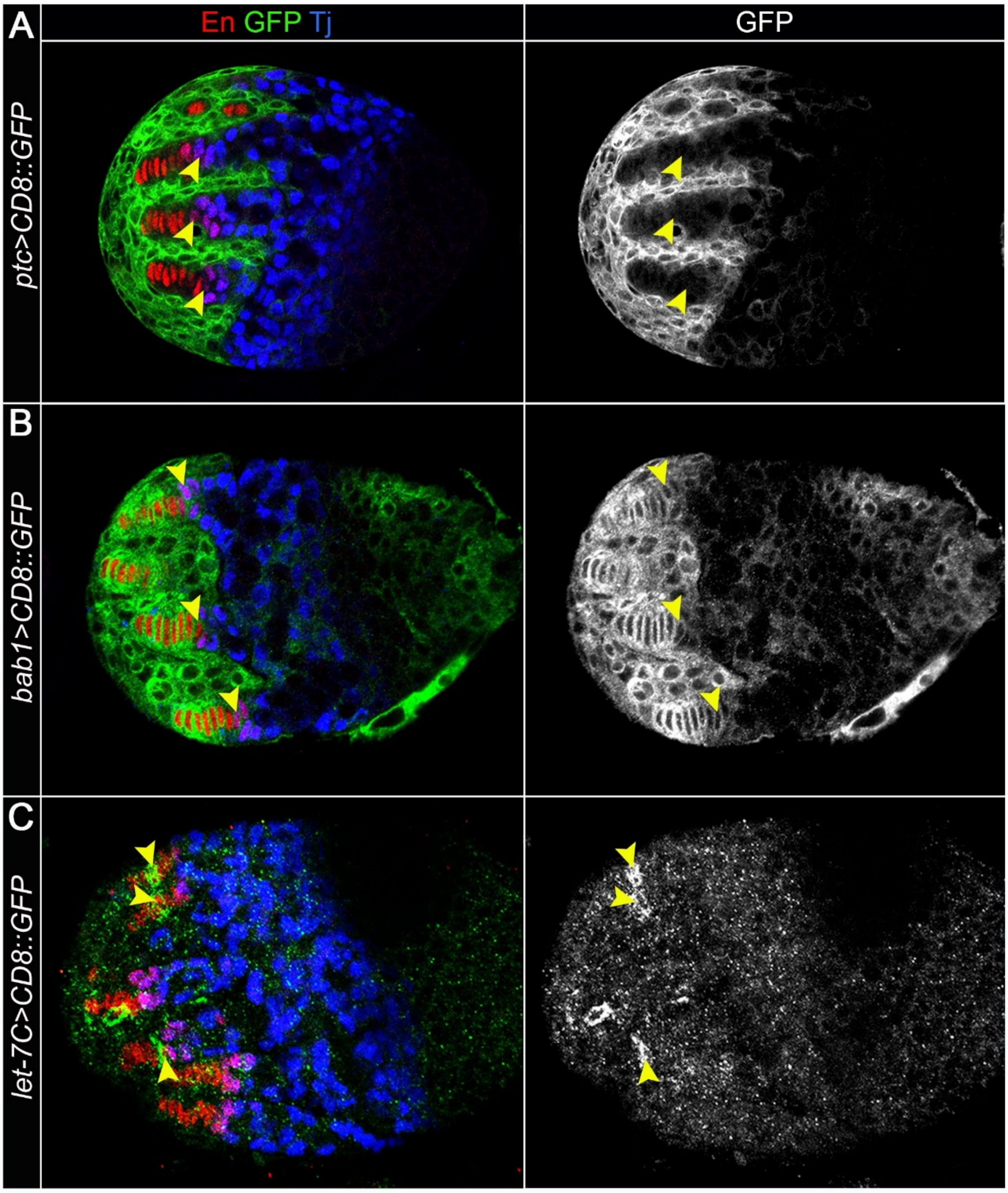

Figure S3. Expression patterns of the used *Gal4* driver lines

A-C Expression patterns of the *Gal4* driver lines crossed to *UAS-CD8::GFP* in the pepupal ovaries. *ptc-Gal4* (B) driver is expressed in ISCs, *bab1-Gal4* (C) driver is expressed in TFCs and CpCs, and *let-7C-Gal4* (D) is expressed in solitary TFCs. All three drivers in addition are expressed in SHCs. GFP (B-D, green, B`-D`, white), En (red) marks TFC, Tj (blue) marks ESCs and Transient TFCs (TCs). TCs and CpCs express both Tj and En (magenta). Arrowheads point to TCs.

Supplementary Figure 4

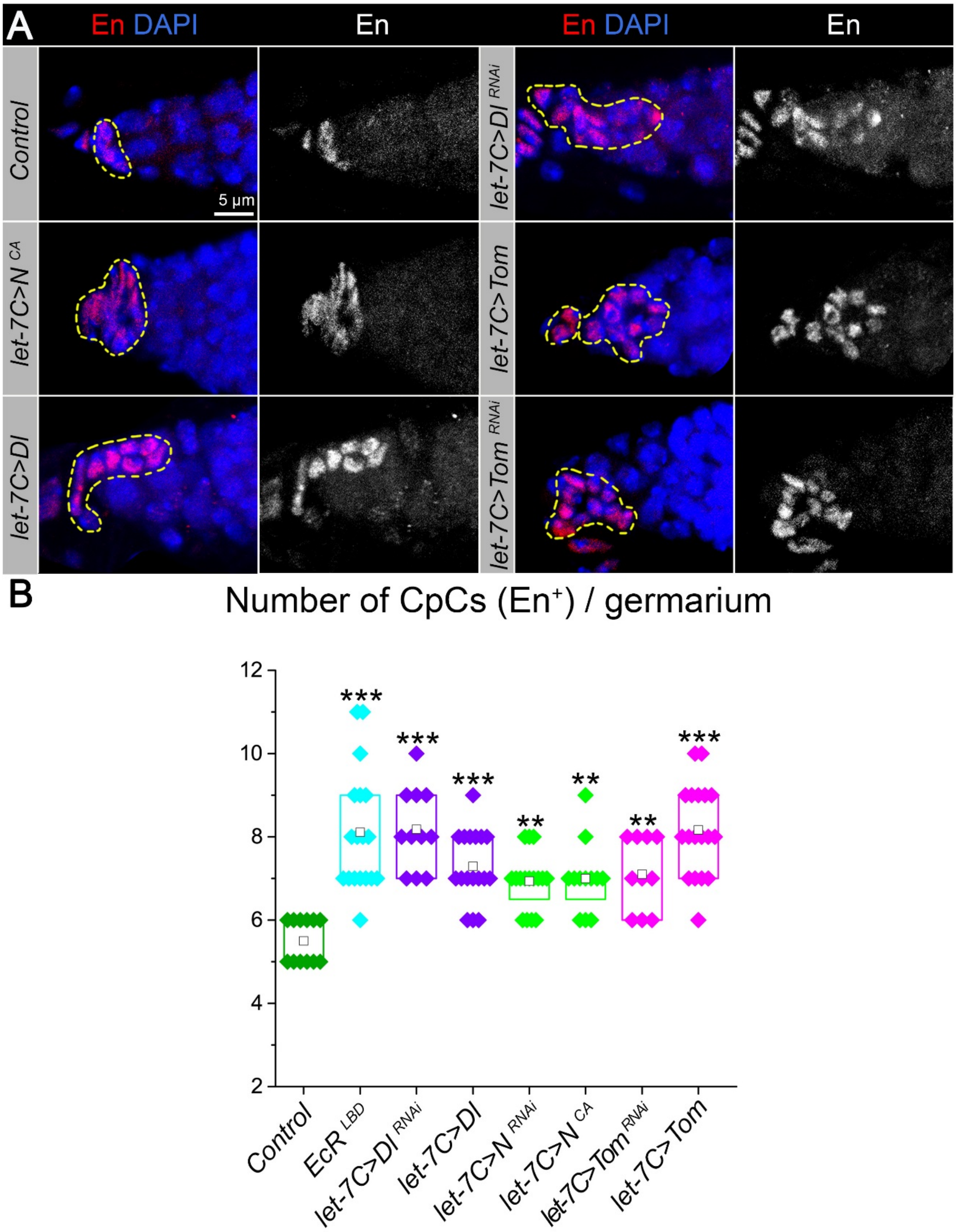

Figure S4. Deregulation of ecdysone, Delta, Notch or Tom affects the GSC niche formation

A. Representative images of the adult germaria with enlarged GSC niches in the Tom-Delta-Notch signaling cascade mutants. Deregulation of Notch, Delta and Tom leads to the increased numbers of CpCs (En, red) and the increased niche size (yellow dashed lines) in comparison to *Control*.

B. Deregulation of ecdysone, Delta, Notch or Tom leads to the increased CpC numbers. Box plots represent quantitative analysis of CpCs per germarium. One-way ANOVA with post-hoc Tukey HSD was used to test for statistical significance: ***P≤0.001, **P≤0.01.

Development • Supplementary information

Supplementary Figure 5

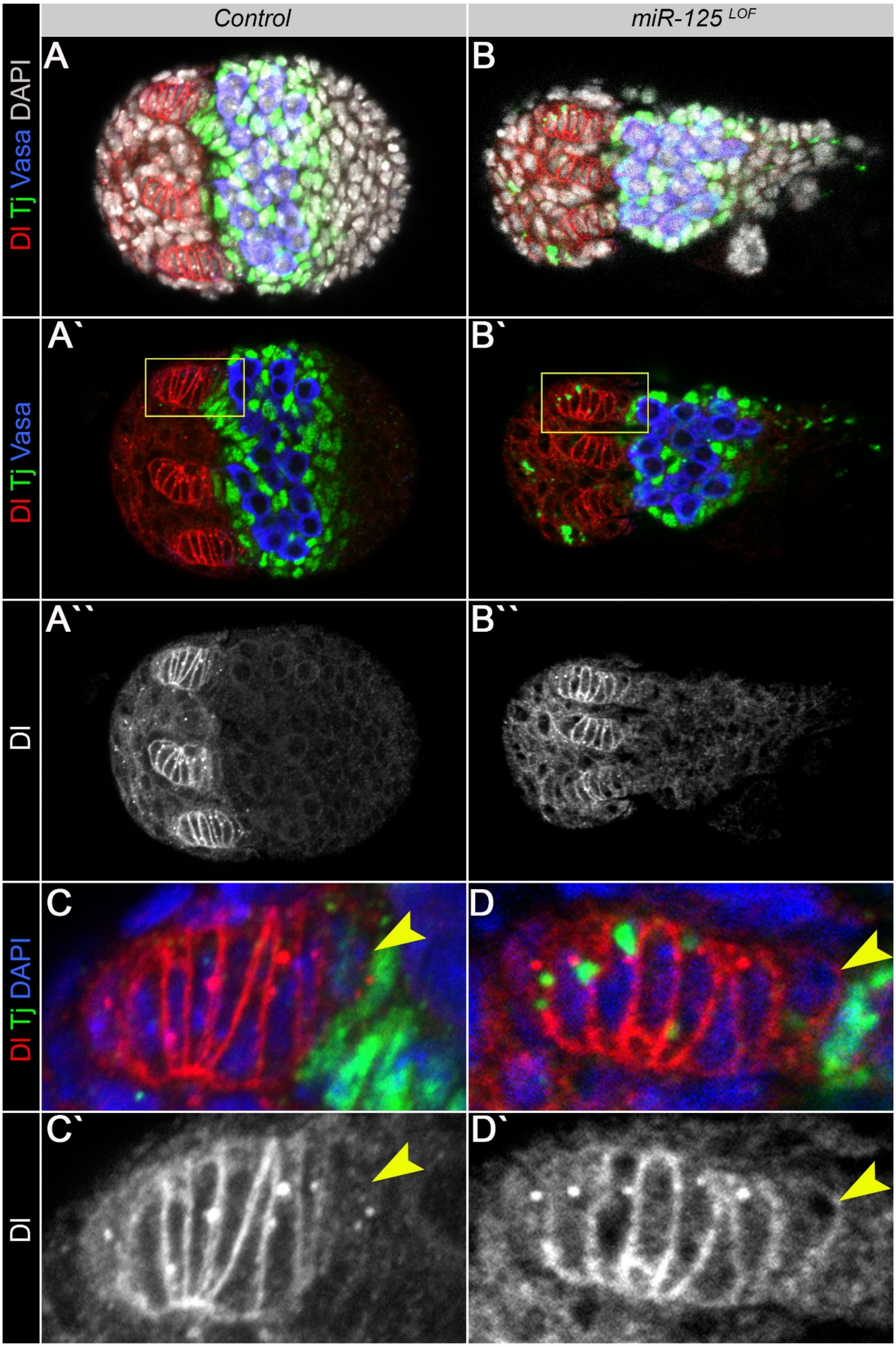

Figure S5. Delta expression is altered in the posterior TFCs of *miR-125* mutants

A-B. Developing ovaries of *Control* and *miR-125* mutants of the comparable stage (Prepupa) shows that the expression pattern of Delta in is not globally altered. **C-D.** However, in the posterior TFCs, the membrane Delta levels are not reduced and the Delta-positive vesicle did not appear in *miR-125* mutants in comparison to controls.

Development • Supplementary information

Supplementary Figure 6

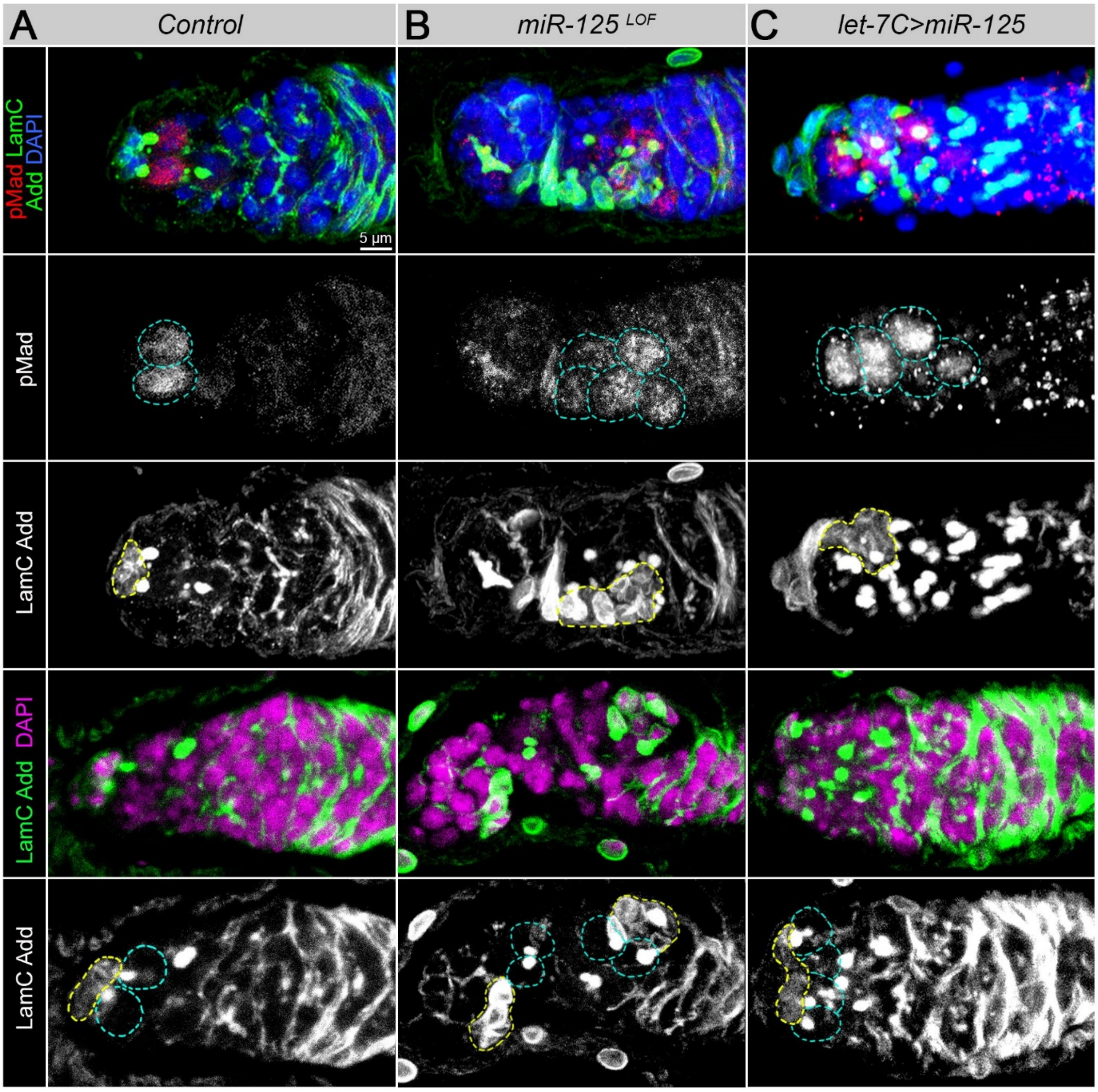

Figure S6. *miR-125* is required for the proper GSC niche establishment

A. Examples of control germaria in controls showing typical adult GSC niches (LamC-positive cells outlined by yellow dashed lines), which normally facilitate two or three GSCs (outlined by cyan dashed lines and marked by pMad and the presence of Add-positive spectrosomes).

B. Loss of *miR-125 (miR-125^{LOF}*) leads to the formation of the ectopic niches (dislocated from the anterior tip), which host supernumerary GSCs.

C. Upregulation of *miR-125* (*let-7C>miR-125*) leads to the enlarged niches, which also can facilitate supernumerary GSCs.
